# Affordability of nutritious diets in rural India

**DOI:** 10.1016/j.foodpol.2020.101982

**Published:** 2021-02

**Authors:** Kalyani Raghunathan, Derek Headey, Anna Herforth

**Affiliations:** a Poverty, Health and Nutrition Division, International Food Policy Research Institute, New Delhi, India; b Poverty, Health and Nutrition Division, International Food Policy Research Institute, Yangon, Myanmar; c Independent Consultant, Boston, MA, United States

**Keywords:** India, Cost of diet, Nutrition, Rural, Affordability

## Abstract

In 2015–16 some 38% of preschool children in India were stunted, 21% wasted, and more than half of Indian mothers and young children were anemic. Though widely studied, surprisingly little research on malnutrition in India explores the role of diets, particularly the affordability of nutritious diets given low wages and the significant structural problems facing India’s agricultural sector. To explore this we used nationally representative rural price and wage data to estimate the least cost means of satisfying India’s national dietary guidelines, referred to as the Cost of a Recommended Diet (CoRD), and assessed the affordability of this diet relative to male and female wages for unskilled laborers. Although we find that dietary costs have increased substantially for both men and women, rural wage rates increased more rapidly, implying that nutritious diets became substantially more affordable over time. However, in absolute terms nutritious diets in 2011 were still expensive relative to unskilled wages, constituting approximately 80–90% of female and 50–60% of male daily wages. Overall, we estimate that 63–76% of the rural poor could not afford a recommended diet in 2011. Achieving nutritional security in India requires a much more holistic focus on improving the affordability of the full range of nutritious food groups (not just cereals), a reappraisal of social protection schemes in light of the cost of more complete nutrition, ensuring that economic growth results in sustained income growth for the poor, and more timely and transparent monitoring of food prices, incomes and dietary costs.

## Highlights

• Limited research focussing on the affordability of diets in India.
• We model the least-cost means of meeting national dietary guidelines.
• We assess the affordability of this least-cost diet against wage data.
• Diets are highly unaffordable, especially for women.
• Greater focus needed on enhancing affordability of nutritious food groups.

## 1 Introduction

India performs exceptionally poorly on a wide range of undernutrition indicators, exhibiting high rates of stunting (38%), wasting (21%) and anemia (58%) among preschoolers, and underweight (23%) and anemia (53%) among adult women (IIPS, 2015). High prevalence rates combined with a large population mean that India is the single largest contributor to maternal and child undernutrition world-wide. This is accompanied by a rising prevalence of overnutrition, especially in urban areas, with an 8–9 percentage point increase in the prevalence of overweight or obese in adult men and women from 2005–06 to 2015–16 (IIPS, 2015). In recognition of these problems, the current Indian government has invested in a national nutrition strategy, including the 2019 launch of the multi-ministry flagship initiative called the *Poshan Abhiyaan* nutrition mission, the official stated goal of which is a malnutrition-free India by 2022. However, *Poshan Abhiyaan* and other nutrition initiatives face a particularly daunting challenge in rural areas where both poverty and malnutrition rates are especially high.

Dietary quality is recognized as a key factor affecting human nutritional status (UNICEF, 1990), and empirical studies have linked dietary diversity to micronutrient adequacy (Arimond et al., 2010, Ruel, 2002) and dietary risk factors to morbidity and mortality from non-communicable diseases (Grosso, 2019). Healthy diets are also associated with reduced risk of both under- and overnutrition and are recommended as ‘double-duty’ actions (Arimond and Ruel, 2004, Hawkes et al., 2019, Headey et al., 2018). Despite this, the role of diets in explaining poor nutritional outcomes in India has received far less attention than is warranted, with little research focusing on access to healthy diets. Previous work has produced some evidence on this topic specific to India, including on agriculture and diets (Headey et al., 2012, Gillespie et al., 2015), vegetarian diets and child nutrition (Headey and Palloni 2020), and the cost of recommended diets in South Asia (Dizon et al., 2019). A recent study by the Indian Ministry of Finance (MoF, 2020) compared the costs of a limited set of foods to industrial wages, and other work has sought solutions to improving the food environment for fruit and vegetable access in India (Thow et al. 2018).

It is highly likely that affordability is a major barrier to improving diets in India. However, both dietary and retail price data are scarce – in the public domain at least – making it difficult to even characterize the problem of affordability of nutritious diets. Household data from the National Sample Survey Organization (NSSO) Consumption Expenditure Survey is only available at infrequent 5-year intervals, with the last round dating to 2011–12. Our paper fills this gap by estimating of the cost of achieving a diet that meets India’s national food-based dietary guidelines (FBDGs), providing a comparison of this cost to earnings for men and women, and describing how affordability has changed over time, by state. We use rural food retail price data to characterize the least-cost recommended diet. While the data we use has limitations (see below), our methods are simple and provide a template for policy-makers and researchers to track the affordability of nutritious diets using available food price data.

Most people in India depend on markets for food acquisition, especially among the poorest wealth quintiles, for whom landlessness is common and farm sizes are small, making these households ‘net consumers’ of food (Ravallion, 1998). Many rural Indians also live in densely populated areas and therefore in close proximity to food markets. Indeed, estimates from nationally representative data from 2011 to 12 suggest that most households rely solely on market purchases for food, although this varies somewhat by food group, being higher for fruits and vegetables (80.5–95.7 percent), above 70 percent even for staples like rice and wheat, and somewhat lower for dairy (see Statement 7-A and 7-B, NSSO, 2012).

Yet although physical access to markets is relatively good in most of rural India, agricultural production systems and markets in India do not necessarily provide access to affordable nutritious diets. India’s Green Revolution had a strong focus on rice and wheat from the late 1960s onwards, at the cost of millet, gram, lentils and oilseeds production (Kumar, 2019, Gowda et al., 2013, Srivastava et al., 2010). Dairy production and marketing improved in the 1970s (the so-called White Revolution), but many other sectors have fared poorly. Fruit and vegetable production has increased recently on the back of growing demand driven by higher incomes and urbanization, but inadequate investment in horticulture, poor storage and transportation infrastructure, and the persistence of government-imposed regulations on food trade have resulted in very inefficient supply chains, with shortages and gluts both common and acute. Moreover, a wide range of policies – including input subsidies, public food distribution and price controls – continue to bias agricultural activities towards rice and wheat, with significant implications for environmental sustainability as well as nutrition (Pingali et al., 2017). Policy strategies from the 2000s onwards have increasingly tried to address food and nutrition insecurity through reforms to food and nutrition assistance programs and social protection schemes - such as the Midday Meals Scheme in government and government-aided schools and the Mahatma Gandhi National Rural Employment Guarantee Act (MGNREGA) that guarantees 100 days of employment per year to every rural household (Raghunathan et al., 2017). However, whether India’s food system is delivering adequate affordable and nutritious foods has remained understudied.

In light of these concerns, the objective of this paper is to advocate for a simple monitoring metric – the estimation of a least cost diet meeting nutritional standards - to assess the ability of markets to deliver affordable nutritious diets to the rural poor in India. The estimation of least cost nutrient adequate diets has a long history going back to Stigler (1945) and continues to be applied as one approach to assessing the cost of a nutritious diet (Chastre et al., 2009, Deptford et al., 2017),^1^ although nutrient-based costing approaches have important conceptual and technical shortcomings (discussed below). Our approach instead costs diets recommended by national nutrition authorities as per methods used in recent studies for South Asia (Dizon et al., 2019), Myanmar (Mahrt et al., 2019), and globally (Herforth et al. 2020).

In the Indian context we define a nutritious diet as one meeting India’s national dietary guidelines (National Institute of Nutrition 2011). Tracking the affordability of a nutritious diet over India’s diverse regions requires high-frequency, spatially disaggregated and representative price and income data. We were fortunate to obtain prices for 101 food items, and wages for various common rural occupations by gender, collected over almost 400 of India’s rural districts on a monthly basis over 2001–2011.^2^ This time period constituted a period of rapid economic growth, significant food inflation, and several major policy developments in the rural economy such as the roll out of MGNREGA from 2005 onwards. We use these price data to cost out the cheapest means of achieving the recommended diet, to examine which foods this diet frequently selects, and to describe how the cost and components of this diet change over time, by gender, and by season. We then compare the cost of this diet to hours-adjusted wages of male and female unskilled laborers, which can be viewed as reservation wages for the poor, especially in a country where millions of landless laborers and marginal farmers earn most of their income from selling their labor (Deaton and Drèze, 2002).^3^ Finally, we examine how the affordability of the recommended diet has varied over time and explore heterogeneity in costs and affordability across India’s diverse states. To the best of our knowledge, no previous published research in India has assessed the cost of a nutritious diet relative to wages with such spatially and temporally rich data.

Since the purpose of this paper is to advocate for the use of price data as a monitoring tool, we rely on market price data rather than the data collected in nationally representative household surveys such as the NSSO’s Consumption Expenditure Survey. Given the focus on monitoring, we would argue that the use of frequent time-series data on prices improves upon rather infrequent waves of household survey data. A major constraint to scaling up the approach used in this paper is the fact that updated disaggregated retail price data for India are no longer in the public domain: our data ends in 2011. However, if and when the more recent waves of this data are released, our method can be easily applied to construct up-to-date estimates of dietary costs and affordability for both rural and urban populations. Government agencies that collect price data can also apply this method to track the cost of meeting national dietary recommendations. The results have useful applications for food and nutrition policies, agricultural and employment policies, and the design of more nutrition-oriented social protection programs. Given the extensive and multidimensional nature of undernutrition in India, more effective approaches to improve access to sufficient, safe and nutritious diets are urgently needed.

## 2 Data and methods

### 2.1 Data

Information on food prices and wages is taken from the Rural Price and Wage Data (Unit Level) – Schedule 3.01 dataset, collected by the National Sample Survey Organization (NSSO) of the Ministry of Statistics and Program Implementation of the Government of India. This dataset collates market-level data at the district level. Price data is available at a monthly frequency for 24 states, 380 districts and 101 food items over the period October 2001 to June 2011.^4^ In total these data encompass 1,772,228 item-month-district observations. However, the panel is not balanced: not all food items are reported in every district (presumably because not all markets in a district sell the full range of 101 products), not all districts appear in each time period, and there are India-wide gaps in data reporting for October 2007 to June 2009.^5^ Hence in total, we have 32,373 distinct district-food item combinations, and 32,743 distinct district-month combinations. Most food items are reported in standard units of grams, kilograms, or liters.

The wage data covers the same time period and geography as the price data. It reports normal daily hours of work and cash and in-kind wages for men, women and children across 18 distinct occupations/activities of varying skill levels.^6^ However, since we are interested in using wages as a proxy for the reservation wages of the poor, we use unskilled labor as our benchmark and retain adult (male and female) cash wage information only for this occupation. In total, we have 5621 distinct district-occupation combinations, and 32,070 distinct district-month combinations.

Two adjustments have been made to these data series. First, wages and prices have been deflated using the Consumer Price Index for Agricultural Laborers (CPI-AL). The CPI data was not available for the newer states of Chhattisgarh, Jharkhand and Uttarakhand that were formed in the early 2000s. To complete the series, therefore, we have used the numbers from the ‘parent states’ of Madhya Pradesh (for Chhattisgarh), Bihar (for Jharkhand) and Uttar Pradesh (for Uttarakhand). Second, since unskilled laborers may not find work for a full 8 hours a day, we have adjusted all wage data by the normal daily hours of work in that district-year-month period to construct a location- and time-specific measure of “expected wage earnings”, (assuming that a full day consists of 8 hours of work). Fig. A.1 plots the trends in these hours for both men and women. We see the normal daily hours for men is close to 8 and displays little variation across season or time. The normal daily hours of work for women are lower at between 5 and 6 h per day, with considerable seasonality, and a slight downward trend overall. For simplicity, we use “earnings” or “hours-adjusted wages” to indicate expected wage earnings.

### 2.2 Methods

#### 2.2.1 Cost of recommended diet

The cost of a nutritious diet can be calculated in various ways depending on how the diet is defined. For decades, there have been attempts to find the lowest cost of meeting nutrient needs through linear programming (Chastre et al., 2009, Deptford et al., 2017, George and Shively, 2017, Håkansson, 2015, Masters et al., 2018, O’Brien-Place and Tomek, 1983, Stigler, 1945). While these methods are useful in highlighting the most nutrient-dense foods per unit of currency and which nutrients add to the cost of the diet, the focus on nutrients has several weaknesses. First, it can result in relatively unrealistic or unpalatable least-cost diets. Second, nutrient density alone does not satisfactorily describe the health properties of foods and diet patterns in protecting health, given the importance of non-nutrient bioactive components of food including fiber, antioxidant and other phytochemicals, and the food matrix. Third, consumers make dietary choices over food groups rather than nutrients (which are largely unobservable or unknown to a typical consumer).

The approach followed in this paper uses India’s FBDG, outlined in the *Dietary Guidelines for Indians* (National Institute of Nutrition 2011), to define a nutritionally adequate, recommended diet. FBDGs are developed to define culturally appropriate diet patterns that meet nutritional needs and protect health, produced and adopted by national governments (Herforth et al., 2019). As such, they constitute national policy and are often the basis for designing nutrition policy and programs. Therefore, the minimum cost of meeting a national FBDG is a highly policy-relevant metric that can lead to insights around which parts of a nutritious diet are most or least affordable over time.

We use a price-based index to measure the Cost of Recommended Diet, or CoRD. This index is a measure of the minimum amount it would cost to meet national FBDG (Herforth et al., 2020), assuming that the entire diet is purchased from the market and not self-provisioned from homestead cultivation or from the commons.^7^ India’s FBDG provides information on the classification of foods into food groups, the recommended serving size and the minimum and maximum number of servings per food group. Somewhat unusually, it also provides gender- and activity-specific serving size recommendations, allowing us to calculate the CoRD separately for men and women engaged in sedentary, moderate or strenuous activities. For the purpose of index construction, we assume that unskilled labor is a moderate activity.

India’s FBDG lists six food groups in total: cereals, proteins (pulses, fish, meat, eggs), dairy, fruit, vegetables, and fats and oils. It does, however, require that one of the vegetables consumed be a dark green leafy vegetable, so for ease of understanding we depict that as a separate food group in this paper. The recommended serving sizes and number of servings for each of these groups are provided in Table 1; men and women differ in recommended servings of staples, proteins and oils and fats.

**Table 1:** Recommended serving sizes and number of servings per day in India's Food-based Dietary Guidelines.

	Serving size (in grams)	Recommend number of servings per day (moderate activity level)		Recommended number of grams per day (moderate activity level)	
		Men	Women	Men	Women
Cereals	30	15	11	450	330
Proteins (pulses, meat, fish, eggs)	30	3	2.5	90	75
Dairy	100	3	3	300	300
Fruit	100	1	1	100	100
Vegetables	100	2	2	200	200
Dark green leafy vegetables	100	1	1	100	100
Oils and fats	5	6	5	30	25

Source: Dietary Guidelines for Indians: A Manual (2011).

The steps followed in the construction of CoRD are as follows:

1. Each of the foods in the price data is classified into one of the food group categories.
2. In the case of multiple types of the same food, only the lowest cost duplicate item is retained. For example, in the case of India, wheat (coarse) and wheat (medium) are both classified simply as wheat, and the more expensive of the two is dropped.
3. All item units are standardized to kilograms. For those items that were in non-standard units (e.g. “a dozen eggs”), estimates of the standard weight of these items were employed.^8^
4. All item prices are converted into price per edible serving, using a price conversion factor that was estimated as follows:

$$\mathrm{price}\;conversion\;factor=\frac{\mathrm{serving}\;size\;\left(in\;g\right)}{\mathrm{price}\;unit\;of\;food\;item\;(in\;g)}/edible\;portion$$

5. For all food groups except dark green leafy vegetables, the two cheapest items in the food group are selected; for dark green leafy vegetables, the cheapest item is chosen.
6. The recommended number of servings for each group and gender is multiplied by the average price per serving for each food group to generate the cost of that food group.
7. Finally, the cost of all food groups is summed to generate CoRD.

Following these steps, we calculate the CoRD separately by gender for each district-month combination that appears in our data. Very high and very low prices are assumed to be anomalies, so we winsorize our data accordingly.

#### 2.2.2 Affordability of diets

We are interested in the relative cost of the recommended diet and need to develop a measure of its affordability. To do this, we calculate a gender-district-time-specific “CoRD/wage ratio” by dividing CoRD by the expected cash wage earnings for men and women in that time period and district (expressed in percentage terms). Similar to the price data, we winsorize very high and very low wage earnings estimates, since these are likely to be erroneous entries.

We then estimate the share of the population that cannot afford CoRD (a poverty headcount measure) and the average gap between the incomes of the CoRD-poor and a CoRD-based poverty line (a poverty gap measure) at the national level. To do this, we use the World Bank’s POVCAL tool which allows researchers to set alternative poverty lines;^9^ fortunately for India this tool disaggregates poverty estimates for rural and urban in India in mid-2011 towards the end of our sample. The cost of a recommended diet will be lower for children due to their lower caloric needs; therefore, adult CoRD estimates over-estimate dietary costs for children. POVCAL does not account for differences in intrahousehold food or non-food expenditure requirements, so we make conservatively low assumptions about non-food expenditure requirements to balance the overestimate of children’s CoRD. A lower benchmark uses the simple but unrealistic assumption that households spend all their income on food. A second benchmark uses the (somewhat arbitrary) assumption that avoiding $1.90/day poverty requires a household to spend at least one-third of its income on non-food expenditures.

#### 2.2.3 Seasonality of cost and affordability of diets

We conduct tests for seasonal differences in food group prices, overall dietary costs, earnings and CoRD/earnings ratios. Specifically, we compute the log of the price/earnings ratio for a given month and regress this against 11 monthly dummy variables (with January as the omitted base category), along with a flexible cubic time trend and district fixed effects. The coefficients on the monthly dummy variables approximately measure the percentage difference in price/earnings ratios between January and all other months of the year.

## 3 Cost and affordability of nutritious diets in rural India

In this section we first report basic descriptive statistics on the nature of the food price data and dietary costs by food group, before examining the composition of the least cost diet, trends in the cost of the recommended diet, seasonality in food prices and the affordability of the recommended diet relative to unskilled hours-adjusted wages and minimum MGNREGA wages.

### 3.1 Prices and dietary costs by food group

Table 2 reports summary statistics for the price data by the seven food groups that make up the recommended diet. The first column reports the number of specific food items in each group. Most are well populated although there are relatively few types of dairy products, fruits and dark green leafy vegetables. Importantly, there are a wide range of cereals, reflecting regional diversity in key staples in India, and a wide range of proteins, including a variety of pulses, which are a key component of the Indian diet.

**Table 2:** Descriptive statistics for the NSSO Rural Price and Wage 2001–2011 dataset by food group.

	Number of food items	Number of price observations (district-month)	Mean price/KG (2011 rupees)	Median price/KG (2011 rupees)	Minimum price/KG (2011 rupees)	Maximum price/KG (2011 rupees)
Cereals	23	32,743	10.3	7.8	0.6	125.4
Proteins (pulses, fish, eggs)	30	32,741	33.8	22.0	5.5	251.7
Dairy	4	32,201	13.4	9.8	2.3	58.0
Fruit	6	32,330	11.8	8.4	1.9	90.9
Vegetables	21	32,733	8.1	6.2	1.2	35.8
Dark green leafy vegetables	5	29,359	6.3	4.7	1.4	22.3
Oils and fats	12	32,614	71.0	48.6	15.0	332.8

Source: Authors’ estimates from the NSSO Rural Price and Wage 2001–2011 dataset. See text for details.

Although the least cost recommended diet metric only draws the cheapest two items from each food group, the prices per kilogram in Table 2 emphasize the extent to which prices vary both within and across food groups, and the scope for consumers to shift towards costlier foods as incomes and food budgets increase.

**Fig. 1:**
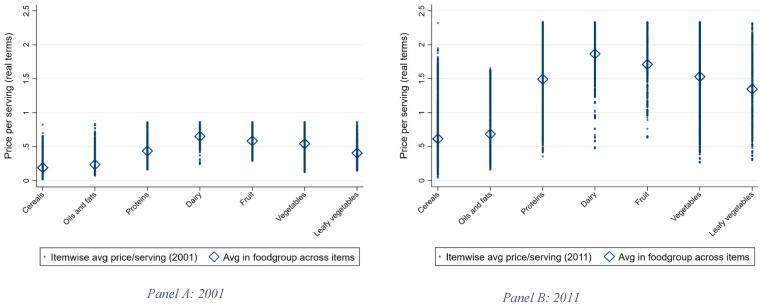
Real food prices per edible serving by food group for rural India in 2001 and 2011 (in 2011 rupees). Source: Authors’ estimates from the NSSO Rural Price 2001–2011 dataset. See text for details.

Fig. 1 shows the average price per serving by food group (converted from price/kg) as well the maxima and minima, with the latter being important in terms of the least cost items. Dairy and fruit are the most expensive food groups per serving, both looking at average and least-cost prices, while oils and fats and cereals are the cheapest. It is clear from the figure that both the average and the dispersion of price per serving have increased substantially between 2001 and 2011.

In Table 3 we report the five most common cheapest food items selected in each food group for CoRD over the full range of observations. Among staples, rice and wheat are often selected, but so too are coarse grains common in some parts of India, such as *bajra* (pearl millet). Because of their lower cost, pulses are much more commonly selected sources of protein than meat, fish or eggs, which offer high-quality protein and various micronutrients, but which are generally much more expensive in kilogram or per calorie terms. In fact, flesh foods and eggs are only selected in 0.02 percent of cases.^10^ Cow and buffalo milk are the two most common dairy items, while tropical fruits dominate the fruit category. Radish and onion are the two most common non-leafy vegetables and *palak* (spinach) is the most commonly selected dark green leafy vegetable. The oils and fats category is populated by a range of oils, including the increasingly popular (and cheap) palm oil. Since availability can vary considerably across geography, Table A.2 presents the most common items in each food group in each state.^11^

**Table 3:** The five most common food items in CoRD (nationally) based on total district-year observations.

Food group	Most common items appearing in CoRD
Cereals	Maize, Bajra, Rice (coarse), Wheat (coarse), Bread
Proteins (pulses, fish, eggs)	Dried peas, Gram, Pea dal, Khesari dal, Gram dal
Dairy	Milk (cow), Milk (buffalo), Curd, Ghol (lassi)
Fruit	Banana, Guava, Papaya, Pineapple, Orange
Vegetables	Radish, Onion, Gourd, Pumpkin, Tomato
Dark green leafy vegetables	Palak, Bhaji sag leaves, Amaranth (chaulai), Mustard leaves, Gogukura
Oils and fats	Mustard oil, Refined oil, Groundnut oil, Palm oil, Gingelly oil

Source: Authors’ estimates from the NSSO Rural Price and Wage 2001–2011 dataset. See text for details.

### 3.2 Trends and variation in prices, dietary costs, wage earnings and dietary affordability

**Fig. 2:**
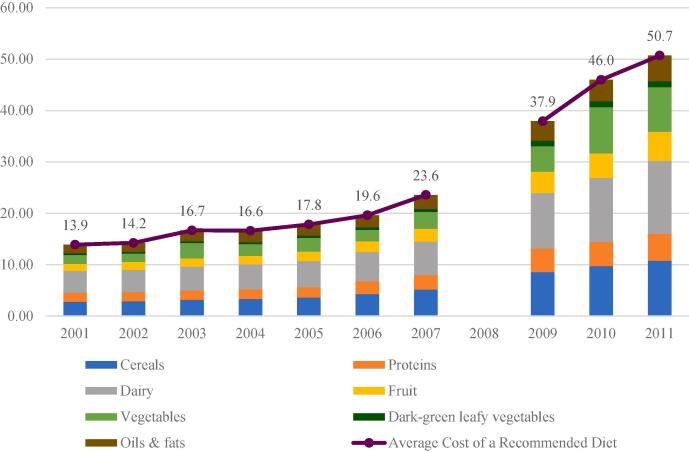
Food group-wise contribution to CoRD based on expected servings, 2001–2011.

Table 4 reports trends in cost per serving for each food group, total dietary costs and male and female unskilled wage earnings over 2001–2011 (all in real terms), as well as the average costs over this period and the coefficient of variation to capture volatility in dietary costs and affordability. Strikingly, real food costs increased dramatically over 2001–2011, albeit with variation across groups. Fig. 2 shows the food group-wise contribution to average CoRD over time.^12^ Dairy is the largest contributor to average CoRD, followed by cereals. Fruits and vegetables as a whole contribute about the same amount to CoRD as dairy (7.2 INR per day on average). Oils and fats contribute the least. There was a sharp increase in CoRD after 2007, coinciding with a period of global food price inflation.

**Table 4:** Trends in cost per serving, wage earnings, CoRD, and CoRD/wage ratios for rural India.

	2001	2011	Average, 2001–2011	Change, 2001–2011	Coefficient of variation, 2001–2011
**Price per recommended consumption per day, in 2011 rupees (cheapest foods)**					
Cereals	2.8	10.8	5.4	8.0	57.4
Oils and fats	1.7	5.0	2.8	3.3	40.3
Fruit	1.4	5.7	2.7	4.3	57.5
Vegetables	1.7	8.6	3.9	6.9	69.2
Dark green leafy vegetables	0.3	1.2	0.6	0.9	56.9
Dairy	4.3	14.2	7.3	9.9	51.2
Proteins (pulses, fish, eggs)	1.8	5.2	2.9	3.4	47.5
**Daily cost of recommended diet (CoRD) in 2011 rupees**					
Male CoRD (INR/day)	14.0	51.3	23.8	37.3	57.5
Female CoRD (INR/day)	12.5	45.1	20.9	32.6	56.4
**Expected wage earnings in 2011 rupees**					
Male expected wage earnings (INR/day)	27.0	106.8	47.5	79.8	132.7
Female expected wage earnings (INR/day)	13.9	52.9	24.7	39	95.4
**Affordability of CoRD relative to expected wage earnings**					
CoRD as % of male expected wage earnings	55.2	54.4	57.9	−0.8	219.3
CoRD as % of female expected wage earnings	84.3	83.5	88.2	−0.8	85.2

Source: Authors’ estimates from the NSSO Rural Price and Wage 2001–2011 dataset. See text for details.

Consistent with rising real food costs, the overall least cost recommended diet increased by 36.4 rupees for men and 31.6 rupees for women over 2001–2011. Over the same period of time India experienced significant economic growth, and wages outpaced real changes in dietary costs, although the change in wage earnings for men was around twice as much as the change for women. Hence, CoRD/wage earnings ratios for men and women both improved somewhat over time, although unaffordability remains strikingly higher for women than men, due to lower wage earnings for women (Table 4, Fig. A.2).

**Fig. 3:**
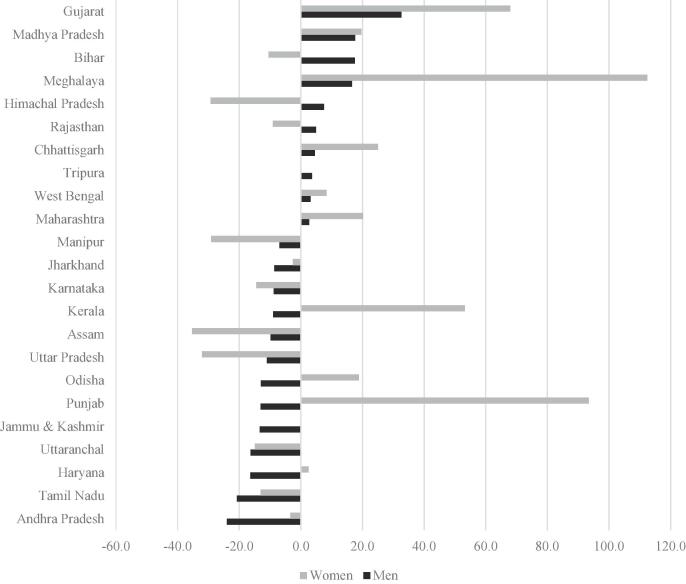
Percent change in CoRD as a fraction of hours-adjusted wages from 2001 to 2011, across states.

Fig. A.3 tracks trends in real wage earnings (hours-adjusted wages) over time for both men and women. Real earnings have increased steeply over the time period of our data, but the rate of increase has been greater for men, especially after 2008. Since national-level numbers hide a lot of variation in a country as large and diverse as India, we also investigate trends in the rate of growth of state-level averages of CoRD/earnings ratios for men and women over 2001–2011, the first and last years in our data (Figs. 3 and A.4, Table A.3). In 2001, male CoRD/earnings ratios were lowest in Kerala (22.9%) – a relatively wealthy state, but also one with strong labor unions and high levels of overseas employment and remittances – as well as Haryana (32.4%), Tamil Nadu (38.8%) and Rajasthan (40.4%), and highest in Chhattisgarh (86.7%), Tripura (77.5%) and West Bengal (68.1%). For women there was much more variation, partly reflecting difference in hours worked: female CoRD/wage ratios were lowest in Kerala, Haryana and Rajasthan (<45%) and highest in Assam (195.1%), Karnataka (139.3%), and Himachal Pradesh (136.3%).

In most states male CoRD/earnings ratios declined over time, suggesting that nutritious recommended diets became substantially more affordable for men, such that by 2011 CoRD/ earnings ratios were typically less than 60 percent (Table A.3).^13^ For women, the patterns are often very different; indeed there is no significant correlation between male and female CoRD/ earnings ratio across states in either 2011 or 2001, or between changes in these ratios over time for men and women. In most states, CoRD/ earnings ratios declined over time for unskilled female workers, but there were some notable exceptions where CoRD/ earnings ratios increased: Gujarat (49.4 pp increase), Punjab (27.1 pp increase) and Kerala (18.5 pp increase). These states saw similar price increases to other states by experienced minimal growth in real wage earnings for women. Gujarat, in particular, has surprisingly high CoRD/ earnings ratios, even in 2011, in spite of relatively rapid economic growth: for Gujarat, CoRD was 122% of expected wage earnings for women and 82.4% for men in 2011.^14^

Next, we provide a comparison of the cost of recommended diet to the wages paid under the large national workfare scheme, the Mahatma Gandhi National Rural Employment Guarantee Act (MGNREGA). Under this Act, notified in 2005 and implemented in early 2006, every rural household is entitled to 100 days of unskilled labor to be compensated at the state minimum wage (subject to a national minimum). The Act guidelines mandate that 33% of jobs be reserved for women, who are to be paid the same wage as men. At the time of its introduction, MGNREGA wages were set at a level higher than prevailing market-determined agricultural and other rural unskilled labor wages, thereby exerting upward pressure on the daily wages of casual laborers (Azam, 2011, Khera, 2011, Zimmermann, 2020, Imbert and Papp, 2015). However, since the entitlement is limited to only a 100 days per household per year, this upward pressure did not necessarily result in an alignment of labor wages with the minimum wages paid under the Act. At the time of the notification of the Act, prevailing unskilled labor wages in 2005 were lower than the MGNREGA wage for both men and women in all states, with the sole exception of male wages in Jammu and Kashmir (Table A.5). Six years later, in 2011, only eight states reported prevailing unskilled labor wages for men greater than the minimum wage, and no state reported unskilled labor wages for women greater than the minimum wage in 2005 or 2011. The difference between the unskilled labor wages and the state-specific minimum wage was often considerable: for men, unskilled labor wages were 28–121% of MGNREGA minimum wages in 2005 and 51–168% in 2011; for women the gap was even larger, at 6–40% in 2005 and 12–88% in 2011 (Table A.5).

**Fig. 4:**
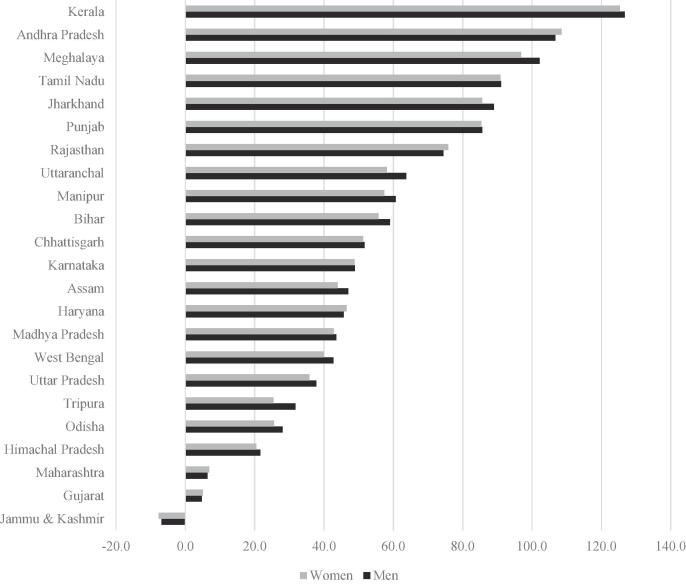
Percent change in CoRD as a fraction of minimum daily wages from 2005 to 2011, across states.

Table A.6 reports CoRD as a percent of state-wise minimum daily wages, which are also the wages paid to workers in the MGNREGA. Since they are indexed to the CPI-AL, it is unsurprising that the MGNREGA minimum wages increased more uniformly across states than the wages for unskilled laborers, and the uniform minimum wage for men and women means that patterns of CoRD/NREGA wage ratios for men and women look similar (since CoRD is similar for men and women). In 2005, CoRD/NREGA wage ratios were relatively low, generally less than 30%, and with a national average of 26.3% for men and 23.3% for women (Table 7). However, the minimum wages for MGNREGA workers, indexed only to inflation, did not increase at the same rate as CoRD, meaning that CoRD/NREGA wage ratios increased by around 50% by 2011, producing a national average of 38.3% for men and 33.6% for women. The largest increases were observed in Andhra Pradesh, Kerala, Tamil Nadu, Jharkhand and Punjab (Fig. 4).

We acknowledge here that while the MGNREGA stipulates state-wise daily minimum wages - regardless of the work being paid on a daily or a piece-rate basis - it is possible that workers are undercut and are not paid the daily minimum. In addition, even though the program is meant to provide work on demand, provision of days of work also varies widely across areas, and often workers do not get the full 100-day allowance specified under the guidelines. Given this, the exercise of comparing CoRD to MGNREGA wages should not be viewed as estimating ‘affordability’ in the same way as the comparison to unskilled labor earnings; rather it is an attempt to estimate the cost of the least-cost diet relative to the ‘best-case’ wage scenario under the program.

### 3.3 Estimating the share of the rural population that cannot afford the recommended diet

Table 5 shows the poverty headcounts and poverty gaps for the rural Indian population if CoRD were used as an alternative poverty line. CoRD for an adult woman amounted to 45.1 rupees per day in June 2011 (in nominal terms). Using the 2011 purchasing power parity conversion factor this amounts to $3.01 per day in international dollars, which is $1.10 above the World Bank’s $1.90/day poverty line (or 58% higher). Adding a 63 cent/day non-food spending requirement (1/3 of expenditures using the $1.90 benchmark) would raise this nutritionally based alternative poverty line from $3.01 to $3.64/day.

**Table 5:** Estimating poverty headcounts and poverty gaps for the rural Indian population in 2011 when CoRD is used in alternative poverty lines.

**Indicator**	**Estimates**
Food and non-food costs	
Cost of Recommended Diet - women, June 2011 (2011 rupees)	45.1
Purchasing power parity conversion factor, 2011 (2011 PPP$)	14.98
CoRD - women, June 2011 (2011 PPP$)	$3.01
Assumed requirements for non-food expenditure (2011 PPP$)	$0.63
Estimate 1 (lower benchmark)	
Rural population unable to afford $3.01 CoRD (poverty headcount)	63.3%
Gap between expenditure of CoRD-poor and CoRD (poverty gap)	20.0%
Estimate 2 (upper benchmark)	
Rural population unable to afford $3.01 CoRD + $0.63 non-food expenditures (poverty headcount)	76.2%
Gap between average expenditure of CoRD-poor and CoRD + non-food spending requirements (poverty gap)	28.7%
World Bank $1.90/day estimates	
Rural population unable to afford $1.90/day expenditures (poverty headcount)	24.8%
Gap between average expenditure of $1.90/day poor and $1.90/day poverty line (poverty headcount)	5.0%

Source: The CoRD estimate in 2011 rupees is drawn from the authors’ estimates from the NSSO Rural Price and Wage 2001–2011 dataset. All other estimates are based on data from the World Bank’s PovcalNet interactive database: http://iresearch.worldbank.org/PovcalNet/povOnDemand.aspx.

The share of the rural Indian population in 2011 unable to afford CoRD even with 100% of income spent on food would be 63.3%, or 527.4 million people. On average, the incomes of that population would be 20% below CoRD. Under the arguably more realistic $3.64 poverty line, 76.2% of the rural population in 2011 could not afford CoRD plus $0.60/day non-food expenditures (634.8 million), and that population’s average income would fall 28.7% below this alternative poverty line. Importantly, these estimates of the share of the rural Indian population that cannot afford the nutritionally recommended diet are much higher than the World Bank $1.90/day estimates of poverty (24.8% of the population identified as poor). These numbers are somewhat speculative, but they do reveal the scale of the dietary affordability problem in rural India: nutritious diets are too expensive, and incomes far too low.

### 3.4 Seasonal variation in prices, dietary costs, wage earnings and dietary affordability

**Fig. 5:**
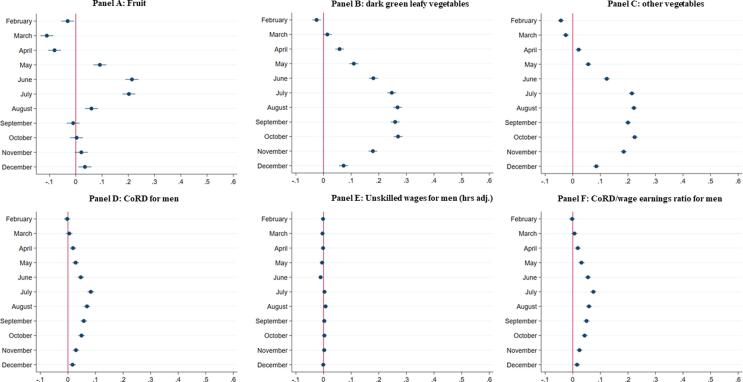
Proportional seasonal changes in fruit prices, vegetable prices, male CoRD, male hours-adjusted wages and male CoRD/wage earnings ratios relative to January. Notes: Authors’ estimates from the NSSO Rural Price and Wage 2001–2011 dataset. See text for details. The figure reports results from multivariate regressions of the log of the differences between the dependent variable’s value for a given month relative to the corresponding value for January. Dependent variables are in nominal terms, and refer to fruit/vegetable prices, CoRD, hours-adjusted wages and CoRD/wage earnings ratios against monthly dummy variables, controlling for linear time trends. Results for CoRD for women, hours-adjusted wages for women and CoRD/wage earnings ratios for women are qualitatively and quantitatively very similar. Results for other food groups show no strong indications of seasonal variation in prices and were therefore omitted for the sake of brevity.

Fig. 5 shows the proportional changes in monthly fruit and vegetable prices (Panel A-C), male CoRD, male wages and male CoRD/wage ratios (Panels D-F) relative to January. These results are presented for the sake of brevity because other food groups showed no statistically or economically significant seasonal variation in prices, and because results for women’s CoRD, hours-adjusted wages and CoRD/wage earnings ratios were almost identical to the results for men in Panels D-F.

Fruit and vegetable prices show very strong seasonality in India. Relative to January, fruit prices fall by 10% in March and April, but then increase rapidly, with June and July prices around 20% higher than January prices. Prices then fall again in the late monsoon (August) and stabilize in the last third of the calendar year. Green leafy and other vegetable prices follow a pattern distinct to that of fruit prices, rising steadily from January until July, at which point they are 25% high than January. Prices remain high from July to October, and then fall sharply down again from November to February. By contrast, the prices of staples, dairy and oils and fats exhibit far less seasonal variation (Fig. A.4).

These increases in fruit and vegetable prices inflate the CoRD. Relative to January, CoRD for men is 3–8% from May through November, with the peak cost coming right in the middle of the monsoon (Panel D). Men’s hours-adjusted wages show no statistically significant seasonality (Panel E). The net result for CoRD/earnings ratios is a 3–7 percent increase from May to November relative to January.

## 4 Limitations of our analysis

The analysis presented above is a stylized comparison of dietary costs relative to expected wage earnings, and while we provide comparisons to per capita income from POVCAL data, we do not account for several additional factors that could affect the calculation of the CoRD and the affordability of nutritious diets. We mention some of these here.

### 4.1 Validity of the retail price data series used

Concerns could be raised about the validity of these price data, especially compared to household-level information obtained from nationally representative consumption surveys such as those conducted by the NSSO. We acknowledge that these retail price data could be unreliable, but household surveys could be as well, and *a priori* there is no reason to expect one source to be more accurate. As a quick check of the validity of these prices, we compared them with wholesale prices from the mandi-level data available from Agmarknet^15^ for a few commonly consumed commodities that could be easily compared. We aggregate the daily mandi-level data to the month level. Fig. A.5 shows national monthly average retail price data (from the NSS-RPC dataset used in the paper) and wholesale price data for a set of commodities chosen as far as possible to provide some representation from each food group. The retail price data is missing from October 2007 to June 2009, but that outside of this time period the two series track each other remarkably well.

### 4.2 Accounting for consumer preferences

The CoRD metric described above cannot explicitly account for consumer preferences the way an analysis of household-level consumption expenditure can. However, consumer preferences do underlie the construction of the country-specific FBDGs, with culturally appropriate diet patterns “baked in” to these guidelines, which is why CoRD baskets typically look more realistic and palatable than calorie-based approaches, which often yield unrealistic and overly monotonous diets. Since our purpose here is to advocate for pricing a diet that adheres to official national nutrition guidelines, we model normative standards for a nutritious diet as developed by the National Institute of Nutrition with a rich understanding of culturally preferred diet patterns in India. Even so, we acknowledge that India’s food cultures are extremely diverse and more refined approaches that factor in household preferences would be an important extension to this strand of research (e.g. Mahrt et al. 2019). When accounting for food preferences, the cost would rise above the least-cost diets that we calculate here.

### 4.3 Accounting for quality and units of purchase

The price data series we use for our analysis does not provide detail on the quality of the food items listed or variations in the price of non-standard units. Both quality and unit of purchase are endogenous to a household’s consumption expenditure decisions (Deaton, 1988) and failure to account for them could introduce approximation errors into our analysis. In particular, the direction of association with household income is not straightforward – while poorer households typically buy lower quality items at lower prices, they will also often be limited to buying smaller quantities at a time, thereby foregoing possible gains from nonlinearities in pricing by unit quantity (such as lower unit prices for bulk purchases). Accounting for this variation could improve the precision of the CoRD estimates but is dependent on the availability of more detailed and granular price data series than the one we use here.

### 4.4 Non-food expenditures and household size

Our analysis looks at the least cost way of attaining an individual diet that meets certain nutritional requirements, however, it does not account for expenditure on non-food items or on the diets of other household members. Since both increase with household size, which is variable by region, we investigate whether state-wise variation in the number of family members is likely to reduce or increase inter-state disparities in affordability of diets. Table A.7 presents the state-wise average rural household size for the set of states in our analysis, as reported in the 2011 Census. For comparison, it also presents the state-wise rank on CoRD to wage ratios for men and women. This table suggests two things. First, that there is some (but not a great deal of) variation in average household size across states. Andhra Pradesh and Tamil Nadu have the smallest households at 3.9 members each, while Uttar Pradesh has the highest at 6 members. The all India average is 4.9. Second, the relationship between household size and CoRD to earnings ratios does not display monotonicity. West Bengal, for example, has a below-national-average household size of 4.4, but is ranked 17 out of 23 in terms of CoRD to earnings ratio; on the other hand, Haryana, which does well on the affordability metric, has an above-national-average household size of 5.4. Correlations between household size and rank on CoRD to earnings ratios for men and women are very low. Given this, we cannot point to a clear relationship between household size and affordability of diets.

### 4.5 Consumption from own production

Our analysis does not account for consumption from own production or from the collection of free foods from common property resources. For rural households, consumption from own production is highest for cereals, pulses and dairy, and even then, a majority of households purchase these foods (NSSO, 2012). Of the set of items NSSO (2012) reports on, the percentage of households relying solely on market purchase in 2011–12 varies across food items, lowest for dairy at 65.5%, and highest for chicken, potatoes and masur dal at more than 95% (see Statements 7-A and 7-B, NSSO 2012). Since fruits and vegetables contribute a large portion of overall costs of the recommended diet and display the greatest seasonality in price variation, consumption from own stocks does not necessarily insulate households from high market prices. On collection of free foods from common property resources, Narayanan (2019) suggests that among the households that engage in this collection (5.8% of all households) these sources play a significant role in reducing the cost of food provision and allowing households to diversify their diets. However, Narayanan (2019) estimates that even for those households, free foods account for only 4.5% of the total value of food consumption. These two sources of food would serve to reduce the cost of achieving a nutritious diet, but the impacts are likely to be modest for most people.

### 4.6 Government social protection schemes

India has in place several social protection schemes that provide cash or in-kind benefits to rural households, with emphasis on the provision of subsidized food and public works programs like the MGNREGA (Dutta et al., 2010, Kapur et al., 2015). Cash transfers such as the maternity benefit scheme, institutional delivery scheme or widow pensions, are relatively recent introductions to India’s social protection landscape, and some, like the maternity benefit scheme, were introduced after the end of our time series. Others provide a one-time payment, as in the institutional delivery scheme, or a paltry amount of around INR 300–500 per month (approximately 4–7 USD) to widows with a below poverty line card, as in the widow pension scheme. In-kind assistance is far more regular and widespread. The PDS provides pre-specified quantities of subsidized rice, wheat and other food items to ration card holders, the Integrated Child Development Scheme (ICDS) provides pregnant and lactating women and young children with supplementary nutrition services, either in the form of hot cooked meals or dry take-home rations, and the midday meal scheme (MDM) provides children aged 6–14 years in government or government-assisted schools with a free lunch. These in-kind benefits serve to reduce out-of-pocket expenditure on food, though evidence of their impact on nutrition outcomes is limited (Raghunathan et al., 2017). We are unable to account for these schemes in our analysis but acknowledge that access to these cash and in-kind benefits could help mitigate high market prices for food. Even so, our main conclusion that the vast majority of rural households in India cannot afford a nutritious recommended diet is almost certainly robust.

## 5 Discussion

For most of India’s post-independence history policymakers have been deeply concerned with achieving basic food security through focusing cereals-oriented agricultural investments, price controls and social safety nets on staple foods. Access to nutritious food has not been a principal focus of food security policy, with the partial exception of dairy. Nutrition has variously been marginalized, regarded as a health problem rather than a complex economic problem, or viewed as a narrow calorie deficiency problem to be addressed by improving the affordability of rice and wheat. Only recently have high level policy initiatives started to envisage strong multisectoral efforts to improve intake of a broader range of nutritious foods to address multiple dimensions of undernutrition and prevent obesity and diet-related non-communicable diseases. However, this invigorated focus on nutrition has entailed remarkably little research on the basic question of how affordable nutritious diets are for India’s poor.

In this study we estimate the cheapest means of achieving nationally recommended diets in rural areas and assessed affordability relative to expected wage earnings for unskilled workers over time and across states. Our study builds on Dizon et al. (2019) – who also cost a recommended diet for South Asia similar to India’s FBGD, but for a recent snapshot only – with data that is much spatially and temporally richer, and with comparisons to a high-frequency series of expected earnings. This approach is also similar to the study by the Indian Ministry of Finance (MoF, 2020), but that study did not cost a diet that adheres to India’s official dietary guidelines; it excluded diary, fruits and dark green leafy vegetables, which are often the most expensive foods in the recommended diet. Moreover, MoF (2020) only compare the cost of their more limited food plate to wages of industrial workers that account for just 5% of the workforce even though the bulk of India’s poor are rural and heavily reliant on the unskilled occupations whose wages we track in the present study. Our study is also similar to that of (Hirvonen et al., 2020), who costed the EAT-Lancet diet for 177 countries for 2011 only, including India. The cost of the EAT-Lancet recommended diet covering 21 food groups is 41 rupees; this is similar in magnitude to the 45 rupees we estimate for women, but somewhat lower than the 51 rupees we estimate for men, though some of this divergence could be a result of differences in the number of calories across the two diets. However, Hirvonen et al. (2020) produced estimates at the national level only, using a different dataset (World Bank ICP) – with the NSSO data, we show there is substantial variation in the cost and affordability of nutritionally recommended diets within India, and across gender.

Given these innovations, what did this study find, and what do our results imply for policy reforms and future research?

### 5.1 Costing nutritious diets and their affordability

A first finding of considerable practical importance is that the approach of calculating least-cost diets based on quantitative food-based dietary guidelines - following Herforth et al. (2020) - results in a realistic selection of food groups that align closely to the mix of foods that Indian households typically consume. In contrast, least-cost diets that satisfy nutrient constraints – following Stigler (1945) – can produce diets that represent a significant departure from existing food preferences. Moreover, India’s national FBDG constitute a policy document, and the minimum cost to access officially recommended diets is therefore an important piece of information for designing policy-coherent actions to improve the ability of the rural poor to consume the recommended diet.

Hence an important policy recommendation is that Indian governments could use this least-cost recommended diet metric to monitor dietary costs and affordability on a timelier and more regular basis, and to consider CoRD as an alternative nutrition-sensitive poverty line. Currently, governments only measure food costs through consumer price indices (CPIs) that weight foods by expenditure shares; however, these expenditure shares in no way reflect the nutritional importance of different foods, and indeed, in poor countries such as India CPIs are heavily weighted towards nutrient-sparse starchy staples, meaning that trends in the food CPI can be misleading from a nutritional standpoint. Furthermore, a practical advantage of CoRD – certainly in comparison to costing nutrient-adequate diets – is its technical simplicity, involving only the identification of the cheapest serving(s) in each food group. Indeed, CoRD can be calculated in a spreadsheet through very basic formulae.

This type of analysis could also be undertaken by non-government organizations and private researchers, although the accessibility of food price data in India is poor, and different price data are collected by different government bodies and released haphazardly or not at all. The NSSO Rural Price and Wage dataset accessed for this study was collected by the Ministry of Statistics and Programme Implementation but has not been updated beyond 2011, contains standardized quality and units of purchase, pertains only to prices from rural centers and was quite challenging to access and use. Urban price data are collected separately by the Labour Bureau, Ministry of Labour and Employment, and are not easily accessible. Quite extensive wholesale price data are collected by the Office of the Economic Adviser in the Department for Promotion of Industry and Internal Trade, but only reported at the national level. Daily wholesale price data at the mandi level is also available from Agmarknet for a large number of commodities, with the prominent exception of dairy. There is, therefore, considerable scope to improve the accessibility of government food price statistics in India, and to use these data sources to analyze food systems more rigorously. Moreover, although CoRD was developed with Indian consumption patterns in mind, future work could apply the “CoRD-Food Preference” method that accounts for local food preferences, developed by Mahrt et al. (2019), to incorporate local consumption patterns within each food group, rather than selecting only least-cost items. Such an exercise is important if there is a “preference premium” by which consumers purchase more expensive foods than is strictly necessary to achieve good nutrition. Such preferences may be especially important in India given its geographical and cultural diversity, as well as the importance of lacto-vegetarianism (Headey and Palloni 2020).

### 5.2 Addressing the rising cost of nutritious diets

We find that nutritious diets are highly unaffordable. We estimated that between 63 and 76% of the rural population of India in 2011 could not afford the recommended diet. Although male and female wages have improved, particularly from about 2007 onwards, recommended diets still accounted for the majority of male wages and in some states still exceeded expected female wage earnings in 2011. It is important to keep in mind that this cost is likely to be an underestimate. It does not allow for individual tastes and preferences, and merely chooses the cheapest items in each food group. It does not take into consideration family size, which, to the extent that the rural poor tend to have larger family sizes, exacerbates the high cost of recommended diets.

### 5.3 Diversifying agriculture toward nutrient-dense foods

We show that the most expensive food groups in this recommended diet are fruits and dairy, and that dietary costs for these two groups rose fastest, although other foods saw real price increases also. The rising cost of these nutrient-dense food groups is perhaps not surprising. Both dairy and fruits have high income elasticities in India (Kumar et al., 2011), such that India’s relatively rapid income growth over 2001–2011 will have significantly raised demand for these products. However, if rising demand is not met by improvements in domestic production and trade then prices may increase commensurately since these highly perishable products cannot cost-effectively be imported from overseas, especially to rural India.

A key goal for nutrition-sensitive agriculture in India must be to raise production, productivity and marketing of more nutritious foods. Previous research has emphasized the need to diversify Indian agriculture largely for the sake of addressing rural poverty, since India’s millions of smallholders need to increase revenue per hectare by switching to higher value agricultural products (Birthal et al., 2013, Joshi et al., 2004, Rao et al., 2006). There are many barriers to diversification of Indian agriculture, including limited public investment in research, development and extension, poor infrastructure (roads/transport, cold storage), and institutional failures that limit smallholders’ access to aggregators and traders, which can be especially problematic for highly perishable products (Pingali, 2015). While these constraints limit the scope for diversification to improve rural incomes, our analysis suggests there is a strong nutritional rationale for using agricultural diversification to curb price inflation for nutrient-dense foods. Furthermore, diversification can increase access to nutrient-dense foods for the rural poor not well connected to markets, where home production or gathering of wild foods may be essential.

### 5.4 Improving rural incomes, especially women’s incomes

Despite the rising cost of nutritious diets over 2001–2011, wage growth for unskilled rural workers typically outpaced rising dietary costs for both men and women, and hours worked declined for women overall. Moreover, several states bucked the generally positive national trend, and there was substantial spatial variation in food affordability trends in rural India over this decade. This result emphasizes the scope for pro-poor economic growth to improve diets, concomitantly with food policy reform to reduce real prices of nutritious foods. It also emphasizes gender inequities in income and in nutritional poverty and highlights the importance of increases in women’s income. We likely underestimate the problem of unaffordability at the household level, because women are often the nutritional gatekeepers for the entire household, including men, and therefore female access to (men’s and women’s) income is likely to affect whether nutritious diets are obtained for the whole household (Doss, 2006, Duflo and Udry, 2004, Hoddinott and Haddad, 1995, Quisumbing et al., 1996).

Since poverty reduction through economic growth is a gradual process, it is also essential to provide income support for the poor. The fact that nutritious diets cost more than international and national poverty lines for India suggests that existing safety net schemes may be inadequate from a nutritional perspective.^16^ Our research, for example, finds that minimum MGNREGA wages cover only one-third of the individual cost of a nutritious diet for a man or woman. This is grossly insufficient given high dependency ratios in rural India as well as significant non-food expenditure requirements. Future research should therefore attempt to understand how India’s various safety net schemes influence diets, but should also assess, ex ante, what level or modality of transfer would suffice to meaningfully improve diets.

### 5.5 Marketing and promotion strategies for healthy dietary diversification

Finally, although improving affordability is a necessary step for improving diets, it is hardly sufficient. Income elasticities for some nutritious foods are high, suggesting that the poor will increase consumption of these foods as incomes rise (Kumar et al., 2011). For other nutritious foods, such as vegetables and pulses, income elasticities are substantially lower, which raises the prospect that income/wage growth will not increase consumption of these foods to desired levels. Moreover, income elasticities for unhealthy processed foods are also high, implying economic growth will dramatically raise consumption of these foods. Indeed, sweet and salty snacks have showed rapid growth in purchases since 2013 (Law et al. 2019). The historical experience of other developing countries suggests that the unhealthy “Westernization” of diets can, if unchecked, proceed rapidly and irreversibly.

A major challenge for nutrition strategies in India is therefore to limit the advertising of and access to unhealthy processed foods, while simultaneously promoting healthy traditional foods and diets. Currently, many nutrition programs target mothers of young children to improve infant and young child feeding practices (understandably), but more effort and innovation is needed to improve consumer knowledge and awareness more generally, such as through media campaigns, school-based interventions and public–private partnerships to improve marketing and advertising for nutritious foods. Furthermore, chemical contamination of vegetables and fruits with pesticide residues or artificial cosmetic enhancement is a growing consumer concern in India (Bailey et al., 2018). For consumer demand to increase for these foods, public trust in food safety is essential, necessitating enforcement of basic food quality standards (Umali-Deininger and Sur, 2007).

Given the persistence of undernutrition in India as well as its rapidly rising rates of obesity and related non-communicable diseases, it is important to reduce prices of nutrient-dense foods on the supply side, and encourage their consumption on the demand side, such that future income growth achieves nutritional dividends. In the meantime, there is also an urgent need to address the widespread unaffordability of the nutritious diets recommended in India’s food-based dietary guidelines through social safety net programs that allow the rural poor to consume sufficient, safe, nutritious foods to meet dietary needs.

## CRediT authorship contribution statement

**Kalyani Raghunathan:** Formal analysis, Writing - original draft, Writing - review & editing. **Derek Headey:** Conceptualization, Writing - original draft, Writing - review & editing, Funding acquisition. **Anna Herforth:** Methodology, Conceptualization, Writing - original draft, Writing - review & editing.

## Footnotes

1 The Cost of the Diet approach has been used by the World Food Programme to conduct ‘Fill the Nutrient Gap’ assessments in several countries, involving various government and non-government stakeholders, although this process has not occurred in India (WFP, 2017).

2 Updated data have not been publicly released.

3 Specifically, Deaton and Drèze (2002) show that changes in unskilled wages over time are a strong predictor of changes in poverty rates, as one would expect from the dependence of India’s poor on selling their labor.

4 India has a total of 28 states and 9 Union territories. The 24 states covered in our dataset account for over 99% of India’s population according to the 2011 Census.

5 The period October 2007-June 2009 saw very steep food price increases because of the global food crisis. Since data are missing for this period, we cannot speak to the effect of the 2007–08 global food crisis on the cost of a nutritious diet, although prices also rose quickly during 2010–11 near the end of our sample.

6 These 18 activities/occupations are unskilled laborer, carpenter, mason, ploughing, blacksmith, sowing, weeding, harvesting, tractor driver, transplanting, cobbler, herdsman, threshing, winnowing, well digging, sweeper, picking (for various crops) and cane crushing.

7 We do not have information on the proportion of unskilled laborers who are also landless. The 18 activities/occupations included in the dataset (see footnote 8) suggest that that an individual classified as an unskilled laborer did not work in agriculture in that given time period. This does not rule out production in a homestead kitchen garden, but it would suggest that the contribution of own production to overall food consumption is relatively low.

9 See http://iresearch.worldbank.org/PovcalNet/povOnDemand.aspx.

10 We should note here that the units for poultry were not provided, and we were unable to make an informed guess at to the approximate weight based on the average prices reported. As a result, poultry had to be dropped from our analysis. We do not believe that this substantially affects the composition of the least cost recommended diet, given that eggs are cheaper than poultry and still only appear in the least cost diet in 0.01 percent of the cases.

11 There is also considerable within-state inter-district variation in the commodities chosen as part of the least-cost commodity basket. Taking Uttar Pradesh as an example (as it has the largest number of districts), we tracked the three most common items in each food group, and found that this selected 8 types of cereals, 10 pulses (no animal-sourced protein was selected), 4 dairy items, 5 fruits, 9 vegetables, 5 dark green leafy vegetables and 9 types of oil or fat. These results are available upon request but have not been presented in the paper due to space constraints.

12 Average CoRD is the CoRD calculated using the expected number of servings, i.e. a simple average of the male and female recommended servings per food group.

13 The largest improvements were in Uttaranchal, Andhra Pradesh and Odisha, where these ratios declined by over 20 percent.

14 It is fairly well known that Gujarat’s economic performance has not translated into high rural or urban skilled or unskilled wages, see for example the India Wage Report 2018 published by the International Labor Organization (https://www.ilo.org/wcmsp5/groups/public/---asia/---ro-bangkok/---sro-new_delhi/documents/publication/wcms_638305.pdf).

15 See agmarknet.gov.in for more information.

16 This result was also found for the United States, which assessed the Supplemental Nutrition Assistance Program (SNAP) benefit against the cost of the federal Dietary Guidelines for Americans (Mulik & Haynes-Maslow, 2017).

## Footnotes

8 There are a few items that are recorded in non-standard units, e.g. a pair (of bananas), each (eggs, oranges), or one (bread). For items such as bananas, eggs and oranges, we make an educated guess as to their approximate weight. For example, each banana is assumed to weigh 120g, a hen egg 44g, a duck’s egg 66g and an orange 131g. For bread, a comparison of the price with current prices in urban Delhi markets suggested that the unit was a 500g loaf. In the case of liquids, we used densities from United States Department of Agriculture (USDA). These densities are given in the Appendix Table A.1.
