# Supplementary material for: Affordability of nutritious diets in rural India
Source: Food Policy. 2021 Feb;99:101982. doi: 10.1016/j.foodpol.2020.101982 (PMC7957322; doi:10.1016/j.foodpol.2020.101982)
Supplement: Supplementary data 1 [file mmc1.docx]

# Appendix

Table A.1: Densities for liquid food items

| Food item | Density (kg/liter) |
| --- | --- |
| Coconut oil | 0.921 |
| Ghol (lassi/buttermilk) | 1.036 |
| Gingelly oil | 0.921 |
| Groundnut oul | 0.913 |
| Linseed oil | 0.926 |
| Milk (buffalo) | 1.031 |
| Milk (cow) | 1.031 |
| Mustard oil | 0.921 |
| Palm oil | 0.913 |
| Rapeseed oil | 0.921 |
| Refined oil (vegetable) | 0.921 |
| Toria oil | 0.921 |
| Source: United States Department of Agriculture (USDA) guidelines. | |

Table A.2: Most common items appearing in CoRD, by state

| **State name** | **Cereals** | **Proteins** | **Dairy** | **Fruit** | **Vegetables** | **Leafy vegetables** | **Oils and fats** |
| --- | --- | --- | --- | --- | --- | --- | --- |
| Andhra Pradesh | Bajra, Ragi, Maize | Peas, Gram, Gram dal | Milk (buffalo), Milk (cow), Curd | Banana, Guava, Papaya (ripe) | Gourd, Onion, Tomato | Gogukura, Palak, Amranth (chaulai) | Palm oil, Groundnut oil (loose), Refined oil |
| Assam | Rice (coarse), Paddy, Bread | Peas, Khesari dal, Gram | Milk (cow), Curd, Milk (buffalo) | Banana, Papaya (ripe), Pineapple | Gourd, Pumpkin, Radish | Bhaji sag leaves, Mustard leaves, Gogukura | Mustard oil (loose), Refined oil, Rapeseed oil |
| Bihar | Maize, Paddy, Wheat (coarse) | Peas, Khesari dal, Pea dal | Milk (cow), Milk (buffalo), Ghol (lassi) | Banana, Guava, Papaya (ripe) | Radish, Gourd, Pumpkin | Bhaji sag leaves, Palak, Amranth (chaulai) | Mustard oil (loose), Refined oil, Ghee (buffalo) |
| Chhatisgarh | Bread, Rice (coarse), Wheat (coarse) | Peas, Khesari dal, Gram | Milk (cow), Milk (buffalo), Curd | Banana, Guava, Papaya (ripe) | Radish, Onion, Pumpkin | Bhaji sag leaves, Palak, Amranth (chaulai) | Refined oil, Mustard oil (loose), Rapeseed oil |
| Delhi | Bajra, Wheat (coarse), Jowar | Gram, Peas, Pea dal | Ghol (lassi), Milk (buffalo), Milk (cow) | Banana, Guava, Pineapple | Radish, Onion, Potato | Amranth (chaulai), Palak, Mustard leaves | Mustard oil (loose), Refined oil, Groundnut oil (loose) |
| Gujarat | Bajra, Maize, Jowar | Peas, Gram, Urd (whole) | Milk (buffalo), Milk (cow), Curd | Banana, Papaya (ripe), Guava | Onion, Potato, Radish | Palak, Bhaji sag leaves, Amranth (chaulai) | Groundnut oil (loose), Mustard oil (loose), Refined oil |
| Haryana | Bajra, Wheat (coarse), Barley | Gram, Peas, Gram dal | Milk (buffalo), Milk (cow), Curd | Banana, Guava, Papaya (ripe) | Radish, Onion, Potato | Palak, Mustard leaves, Bhaji sag leaves | Mustard oil (loose), Refined oil, Toria oil |
| Himachal Pradesh | Wheat atta, Maize atta, Rice (coarse) | Gram dal, Besan (ground gram dal), Urd (whole) | Milk (cow), Curd, Ghol (lassi) | Banana, Guava, Mango | Potato, Onion, Pumpkin | Palak, Gogukura, Amranth (chaulai) | Mustard oil (loose), Ghee mixed (cow & buffalo), Refined oil |
| Jammu & Kashmir | Maize, Bread, Wheat atta | Gram dal, Peas, Gram | Milk (cow), Curd, Milk (buffalo) | Banana, Orange (med), Guava | Radish, Potato, Onion | Palak, Mustard leaves, Bhaji sag leaves | Mustard oil (loose), Ghee mixed (cow & buffalo), Ghee (cow) |
| Jharkhand | Paddy, Maize, Wheat (coarse) | Peas, Khesari dal, Gram | Milk (cow), Milk (buffalo), Curd | Banana, Guava, Papaya (ripe) | Radish, Gourd, Pumpkin | Palak, Bhaji sag leaves, Amranth (chaulai) | Mustard oil (loose), Refined oil, Rapeseed oil |
| Karnataka | Ragi, Rice (coarse), Jowar | Gram, Peas, Besan (ground gram dal) | Milk (cow), Milk (buffalo), Curd | Banana, Guava, Pineapple | Radish, Tomato, Onion | Palak, Bhaji sag leaves, Mustard leaves | Groundnut oil (loose), Refined oil, Palm oil |
| Kerala | Tapioca, Rice (coarse), Ragi | Peas, Gram, Pea dal | Milk (cow), Curd, Milk (buffalo) | Banana, Guava, Pineapple | Onion fresh, Gourd, Pumpkin | Palak, Amranth (chaulai), Bhaji sag leaves | Palm oil, Coconut oil, Gingelly oil (loose) |
| Madhya Pradesh | Wheat (coarse), Maize, Jowar | Gram, Peas, Soyabean | Milk (buffalo), Milk (cow), Curd | Banana, Papaya (ripe), Guava | Onion, Radish, Potato | Palak, Bhaji sag leaves, Amranth (chaulai) | Mustard oil (loose), Refined oil, Groundnut oil (loose) |
| Maharashtra | Jowar, Bajra, Bread | Peas, Gram, Khesari dal | Milk (buffalo), Milk (cow), Curd | Banana, Guava, Papaya (ripe) | Onion, Radish, Tomato | Palak, Bhaji sag leaves, Amranth (chaulai) | Refined oil, Groundnut oil (loose), Palm oil |
| Manipur | Maize, Rice (coarse), Paddy | Peas, Pea dal, Gram | Milk (cow), Curd, Milk (buffalo) | Pineapple, Banana, Guava | Pumpkin, Cabbage, Gourd | Mustard leaves, Palak, Bhaji sag leaves | Palm oil, Refined oil, Mustard oil (loose) |
| Meghalaya | Rice (coarse), Bread, Wheat atta | Peas, Gram, Khesari dal | Milk (cow), Curd, Milk (buffalo) | Banana, Pineapple, Papaya (ripe) | Radish, Sweet potato, Potato | Bhaji sag leaves, Mustard leaves, Palak | Mustard oil (loose), Refined oil, Ghee (cow) |
| Odisha | Bread, Paddy, Rice (coarse) | Peas, Urd (whole), Pea dal | Milk (cow), Milk (buffalo), Ghol (lassi) | Banana, Guava, Papaya (ripe) | Gourd, Radish, Pumpkin | Bhaji sag leaves, Palak, Amranth (chaulai) | Palm oil, Refined oil, Mustard oil (loose) |
| Punjab | Wheat (med.), Wheat atta, Wheat (coarse) | Peas, Gram, Gram dal | Milk (buffalo), Milk (cow), Ghol (lassi) | Banana, Guava, Pineapple | Radish, Potato, Onion | Palak, Mustard leaves, Bhaji sag leaves | Mustard oil (loose), Toria oil, Refined oil |
| Rajasthan | Bajra, Barley, Maize | Gram, Gram dal, Soyabean | Milk (buffalo), Milk (cow), Curd | Banana, Papaya (ripe), Guava | Onion, Radish, Potato | Palak, Amranth (chaulai), Bhaji sag leaves | Mustard oil (loose), Refined oil, Groundnut oil (loose) |
| Tamil Nadu | Bajra, Ragi, Jowar | Peas, Pea dal, Gram | Milk (cow), Milk (buffalo), Curd | Banana, Guava, Papaya (ripe) | Gourd, Pumpkin, Radish | Palak, Bhaji sag leaves, Amranth (chaulai) | Groundnut oil (loose), Gingelly oil (loose), Palm oil |
| Tripura | Rice (coarse), Rice (med.), Wheat atta | Peas, Gram, Pea dal | Milk (cow), Curd, Ghol (lassi) | Banana, Orange (med), Pineapple | Pumpkin, Radish, Cabbage | Bhaji sag leaves, Mustard leaves, Palak | Mustard oil (loose), Refined oil, Rapeseed oil |
| Uttar Pradesh | Wheat (coarse), Bajra, Maize | Peas, Pea dal, Gram | Milk (buffalo), Milk (cow), Curd | Banana, Guava, Papaya (ripe) | Radish, Gourd, Potato | Palak, Amranth (chaulai), Mustard leaves | Mustard oil (loose), Refined oil, Ghee (buffalo) |
| Uttaranchal | Rice (coarse), Wheat atta, Bajra | Peas, Soyabean, Pea dal | Milk (buffalo), Curd, Milk (cow) | Banana, Guava, Orange (med) | Radish, Potato, Cabbage | Palak, Mustard leaves, Bhaji sag leaves | Refined oil, Mustard oil (loose), Palm oil |
| West Bengal | Paddy, Bread, Rice (coarse) | Peas, Khesari dal, Pea dal | Milk (cow), Milk (buffalo), Curd | Banana, Guava, Papaya (ripe) | Gourd, Radish, Pumpkin | Bhaji sag leaves, Palak, Mustard leaves | Mustard oil (loose), Palm oil, Refined oil |

Table A.3: CoRD as a % of unskilled hours-adjusted wages by state, 2001 and 2011

|  | **% of Men’s unskilled hrs. adj. wages** | | | **% of Women’s unskilled hrs. adj. wages** | | |
| --- | --- | --- | --- | --- | --- | --- |
| State name | **2001** | **2011** | **% change** | **2001** | **2011** | **% change** |
| Andhra Pradesh | 59.0 | 44.8 | -24.0 | 83.2 | 80.4 | -3.4 |
| Assam | 67.2 | 60.6 | -9.8 | 195.1 | 126.2 | -35.3 |
| Bihar | 64.3 | 75.6 | 17.5 | 86.4 | 77.3 | -10.5 |
| Chhattisgarh | 86.7 | 90.6 | 4.5 | 81.1 | 101.4 | 25.0 |
| Gujarat | 62.2 | 82.4 | 32.6 | 72.8 | 122.2 | 67.9 |
| Haryana | 32.4 | 27.1 | -16.4 | 43.0 | 44.1 | 2.5 |
| Himachal Pradesh | 48.5 | 52.1 | 7.5 | 136.3 | 96.3 | -29.3 |
| Jammu & Kashmir | 40.0 | 34.7 | -13.3 |  |  |  |
| Jharkhand | 58.5 | 53.4 | -8.6 | 62.3 | 60.7 | -2.6 |
| Karnataka | 60.7 | 55.3 | -8.8 | 139.3 | 119.1 | -14.5 |
| Kerala | 22.9 | 20.8 | -9.0 | 34.7 | 53.2 | 53.2 |
| Madhya Pradesh | 64.2 | 75.5 | 17.6 | 74.1 | 88.7 | 19.7 |
| Maharashtra | 55.3 | 56.8 | 2.7 | 90.5 | 108.8 | 20.2 |
| Manipur | 66.6 | 61.9 | -7.0 | 91.9 | 65.2 | -29.1 |
| Meghalaya | 66.3 | 77.3 | 16.6 | 66.0 | 140.2 | 112.4 |
| Odisha | 57.4 | 50.0 | -13.0 | 60.7 | 72.1 | 18.8 |
| Punjab | 37.0 | 32.1 | -13.1 | 29.1 | 56.2 | 93.5 |
| Rajasthan | 40.4 | 42.4 | 5.0 | 45.9 | 41.7 | -9.1 |
| Tamil Nadu | 38.8 | 30.8 | -20.7 | 74.8 | 65.1 | -13.0 |
| Tripura | 77.5 | 80.4 | 3.6 |  |  |  |
| Uttar Pradesh | 51.5 | 45.8 | -11.0 | 81.8 | 55.6 | -32.0 |
| Uttaranchal | 48.8 | 40.8 | -16.3 | 68.5 | 58.3 | -14.9 |
| West Bengal | 68.1 | 70.2 | 3.1 | 107.7 | 116.6 | 8.3 |

Source: Authors’ estimates from the NSSO Rural Price and Wage 2001-2011 dataset. See text for details.

Table A.4: CoRD and hours-adjusted unskilled labor wages for men and women, 2011

|  | **Men** | | **Women** | |
| --- | --- | --- | --- | --- |
| **State name** | **CoRD (in June 2011 prices)** | **Unskilled labor wages (hours adjusted, in June 2011 prices)** | **CoRD (in June 2011 prices)** | **Unskilled labor wages (hours adjusted, in June 2011 prices)** |
| Andhra Pradesh | 47.19 | 109.21 | 41.97 | 68.08 |
| Assam | 61.10 | 101.99 | 53.13 | 15.45 |
| Bihar | 46.87 | 63.25 | 40.97 | 38.26 |
| Chhattisgarh | 48.06 | 62.80 | 42.68 | 50.67 |
| Gujarat | 52.96 | 70.46 | 46.35 | 41.19 |
| Haryana | 48.93 | 182.39 | 43.62 | 136.02 |
| Himachal Pradesh | 73.21 | 143.02 | 62.75 | 46.30 |
| Jammu & Kashmir | 59.39 | 174.86 | 51.73 | 0.00 |
| Jharkhand | 49.01 | 95.14 | 43.11 | 84.15 |
| Karnataka | 43.41 | 83.35 | 37.87 | 31.81 |
| Kerala | 50.76 | 252.12 | 44.32 | 51.28 |
| Madhya Pradesh | 48.90 | 71.55 | 43.06 | 59.57 |
| Maharashtra | 46.02 | 84.63 | 40.72 | 43.15 |
| Manipur | 53.01 | 85.60 | 46.41 | 76.68 |
| Meghalaya | 73.72 | 99.89 | 61.51 | 39.95 |
| Odisha | 43.40 | 90.40 | 37.77 | 69.88 |
| Punjab | 52.73 | 169.48 | 46.05 | 21.97 |
| Rajasthan | 51.58 | 128.56 | 45.58 | 105.21 |
| Tamil Nadu | 43.75 | 153.85 | 38.61 | 71.39 |
| Tripura | 80.95 | 100.92 | 69.21 | 0.00 |
| Uttar Pradesh | 45.90 | 102.19 | 40.23 | 49.97 |
| Uttaranchal | 59.86 | 146.26 | 51.61 | 27.49 |
| West Bengal | 48.39 | 71.33 | 42.69 | 41.76 |

Source: Authors’ estimates from the NSSO Rural Price and Wage 2001-2011 dataset. See text for details.

Notes: “Hours-adjusted” wages deflate wages by the normal hours of work relative to a ‘full day’, assumed to be 8 hours.

Table A.5: Hours-adjusted unskilled labor wages for men and women as a percentage of state-wise NREGA minimum wages, 2005 and 2011

|  | **Hours-adjusted unskilled labor wages as %age of NREGA wage in 2005** | | **Hours-adjusted unskilled labor wages as %age of NREGA wage in 2011** | |
| --- | --- | --- | --- | --- |
| **State Name** | **Men** | **Women** | **Men** | **Women** |
| Andhra Pradesh | 33.75 | 21.58 | 90.26 | 56.27 |
| Assam | 57.79 | 6.33 | 78.46 | 11.89 |
| Bihar | 43.25 | 21.39 | 52.71 | 31.89 |
| Chhattisgarh | 28.59 | 22.73 | 51.47 | 41.53 |
| Gujarat | 54.16 | 40.79 | 56.82 | 33.22 |
| Haryana | 51.00 | 37.03 | 101.89 | 75.99 |
| Himachal Pradesh | 84.74 | 13.21 | 105.94 | 34.30 |
| Jammu & Kashmir | 121.19 | - | 144.52 | - |
| Jharkhand | 43.29 | 36.78 | 79.29 | 70.13 |
| Karnataka | 39.99 | 21.76 | 66.68 | 25.45 |
| Kerala | 67.21 | 25.83 | 168.08 | 34.18 |
| Madhya Pradesh | 41.17 | 31.60 | 58.64 | 48.83 |
| Maharashtra | 55.58 | 31.57 | 66.64 | 33.97 |
| Manipur | 45.56 | 32.87 | 67.93 | 60.86 |
| Meghalaya | 62.24 | 18.13 | 85.38 | 34.15 |
| Odisha | 47.20 | 38.50 | 72.32 | 55.91 |
| Punjab | 46.60 | 16.19 | 110.77 | 14.36 |
| Rajasthan | 48.82 | 37.95 | 108.03 | 88.41 |
| Tamil Nadu | 50.45 | 29.26 | 129.28 | 59.99 |
| Tripura | 70.55 | - | 85.52 | - |
| Uttar Pradesh | 55.33 | 28.86 | 85.16 | 41.64 |
| Uttaranchal | 62.28 | 19.55 | 121.88 | 22.91 |
| West Bengal | 40.74 | 25.39 | 54.87 | 32.12 |

Source: Authors’ estimates from the NSSO Rural Price and Wage 2001-2011 dataset. See text for details.

Notes: “Hours-adjusted” wages deflate wages by the normal hours of work relative to a ‘full day’, assumed to be 8 hours.

Table A.6: CoRD as a percentage of the Mahatma Gandhi National Rural Employment Guarantee Act (MGNREGA) minimum wages for men and women, by state, 2005 & 2011

| **State name** | **Men’s CoRD/NREGA wage ratios** | | |  | **Women’s CoRD/NREGA wage ratios** | | |
| --- | --- | --- | --- | --- | --- | --- | --- |
|  | **2005** | **2011** | **% change** |  | **2005** | **2011** | **% change** |
| All rural India | 26.28 | 38.30 | 45.75 |  | 23.29 | 33.66 | 44.52 |
|  |  |  |  |  |  |  |  |
| Andhra Pradesh | 18.87 | 39.00 | 106.69 |  | 16.64 | 34.68 | 108.48 |
| Assam | 31.98 | 47.00 | 46.98 |  | 28.40 | 40.87 | 43.92 |
| Bihar | 24.57 | 39.06 | 58.99 |  | 21.93 | 34.14 | 55.68 |
| Chhattisgarh | 25.98 | 39.40 | 51.66 |  | 23.13 | 34.98 | 51.24 |
| Gujarat | 40.77 | 42.71 | 4.75 |  | 35.57 | 37.38 | 5.09 |
| Haryana | 18.76 | 27.34 | 45.69 |  | 16.63 | 24.37 | 46.51 |
| Himachal Pradesh | 44.57 | 54.23 | 21.67 |  | 38.57 | 46.48 | 20.51 |
| Jammu & Kashmir | 52.73 | 49.08 | -6.91 |  | 46.30 | 42.75 | -7.68 |
| Jharkhand | 21.61 | 40.84 | 89.03 |  | 19.36 | 35.92 | 85.61 |
| Karnataka | 23.32 | 34.73 | 48.92 |  | 20.36 | 30.29 | 48.79 |
| Kerala | 14.92 | 33.84 | 126.73 |  | 13.12 | 29.55 | 125.28 |
| Madhya Pradesh | 27.93 | 40.09 | 43.55 |  | 24.72 | 35.29 | 42.78 |
| Maharashtra | 34.05 | 36.23 | 6.41 |  | 30.00 | 32.06 | 6.88 |
| Manipur | 26.18 | 42.07 | 60.69 |  | 23.40 | 36.83 | 57.38 |
| Meghalaya | 31.16 | 63.01 | 102.19 |  | 26.71 | 52.57 | 96.81 |
| Odisha | 27.12 | 34.72 | 28.01 |  | 24.06 | 30.21 | 25.58 |
| Punjab | 18.57 | 34.47 | 85.61 |  | 16.24 | 30.10 | 85.36 |
| Rajasthan | 24.86 | 43.34 | 74.37 |  | 21.78 | 38.30 | 75.85 |
| Tamil Nadu | 19.24 | 36.76 | 91.10 |  | 17.00 | 32.45 | 90.85 |
| Tripura | 52.08 | 68.60 | 31.73 |  | 46.75 | 58.65 | 25.44 |
| Uttar Pradesh | 27.76 | 38.25 | 37.79 |  | 24.69 | 33.53 | 35.78 |
| Uttaranchal | 30.48 | 49.89 | 63.67 |  | 27.21 | 43.01 | 58.09 |
| West Bengal | 26.08 | 37.23 | 42.72 |  | 23.47 | 32.84 | 39.89 |

Source: Authors’ estimates from the NSSO Rural Price and Wage 2001-2011 dataset. NREGA wages in 2005-06 and 2011 were obtained from Ministry of Rural Development, Government of India (<https://nrega.nic.in/nerega_statewise.pdf> ). See text for details.

**Notes:** The NREGA was notified in 2005, so the two years presented here are state-wise minimum wages at the time of notification of the Act and in the last year in our data. Missing blocks come from states where wages for female unskilled laborers were not reported.

Table A.7: Average household size and rank on CoRD to wage earnings ratios for men and women

|  |  | **Rank on affordability in 2011 (CoRD to wage ratios) in decreasing order (most to least affordable)** | |
| --- | --- | --- | --- |
| **State Name** | **Average household size (rural)** | **Men** | **Women** |
| Andhra Pradesh | 3.9 | 8 | 12 |
| Assam | 4.9 | 15 | 20 |
| Bihar | 5.5 | 19 | 11 |
| Chhattisgarh | 4.5 | 23 | 15 |
| Gujarat | 5.1 | 22 | 19 |
| Haryana | 5.4 | 2 | 2 |
| Himachal Pradesh | 4.7 | 11 | 14 |
| Jammu & Kashmir | 5.8 | 5 |  |
| Jharkhand | 5.3 | 12 | 7 |
| Karnataka | 4.7 | 13 | 18 |
| Kerala | 4.2 | 1 | 3 |
| Madhya Pradesh | 4.7 | 18 | 13 |
| Maharashtra | 4.6 | 14 | 16 |
| Manipur | 5.2 | 16 | 9 |
| Meghalaya | 5.5 | 20 | 21 |
| Odisha | 4.3 | 10 | 10 |
| Punjab | 5.2 | 4 | 5 |
| Rajasthan | 5.4 | 7 | 1 |
| Tamil Nadu | 3.9 | 3 | 8 |
| Tripura | 4.4 | 21 |  |
| Uttar Pradesh | 6 | 9 | 4 |
| Uttaranchal | 4.9 | 6 | 6 |
| West Bengal | 4.5 | 17 | 17 |
| **All India** | 4.9 |  |  |

Source: Data on average rural household size is taken from the 2011 Census, <https://censusindia.gov.in/2011census/hh-series/hh01.html>. Data on CoRD to wage ratios is from the NSSO Rural price and wage schedule, see text for details.


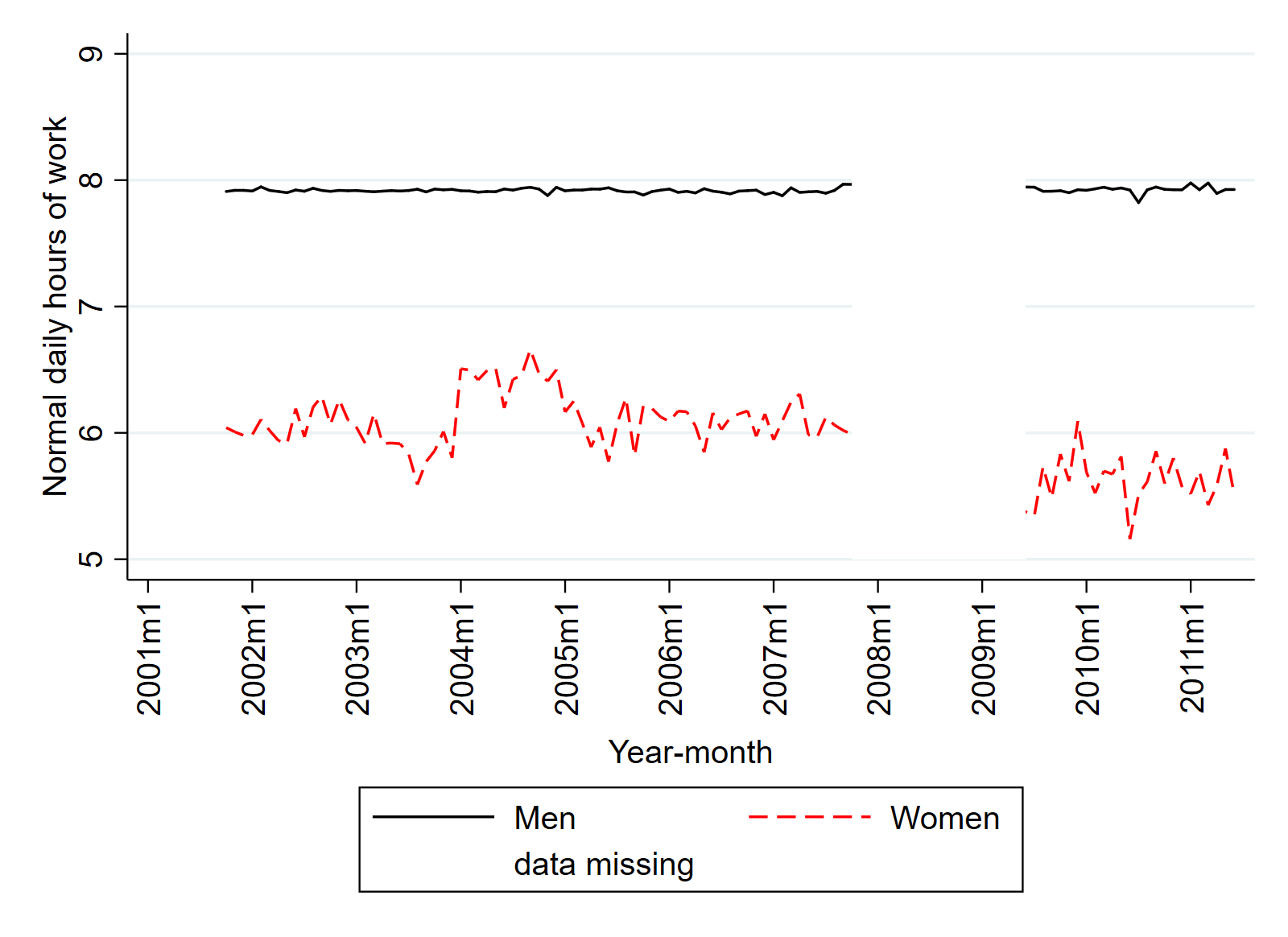


Figure A.1: Trends in normal hours worked for men and women engaged in unskilled labour


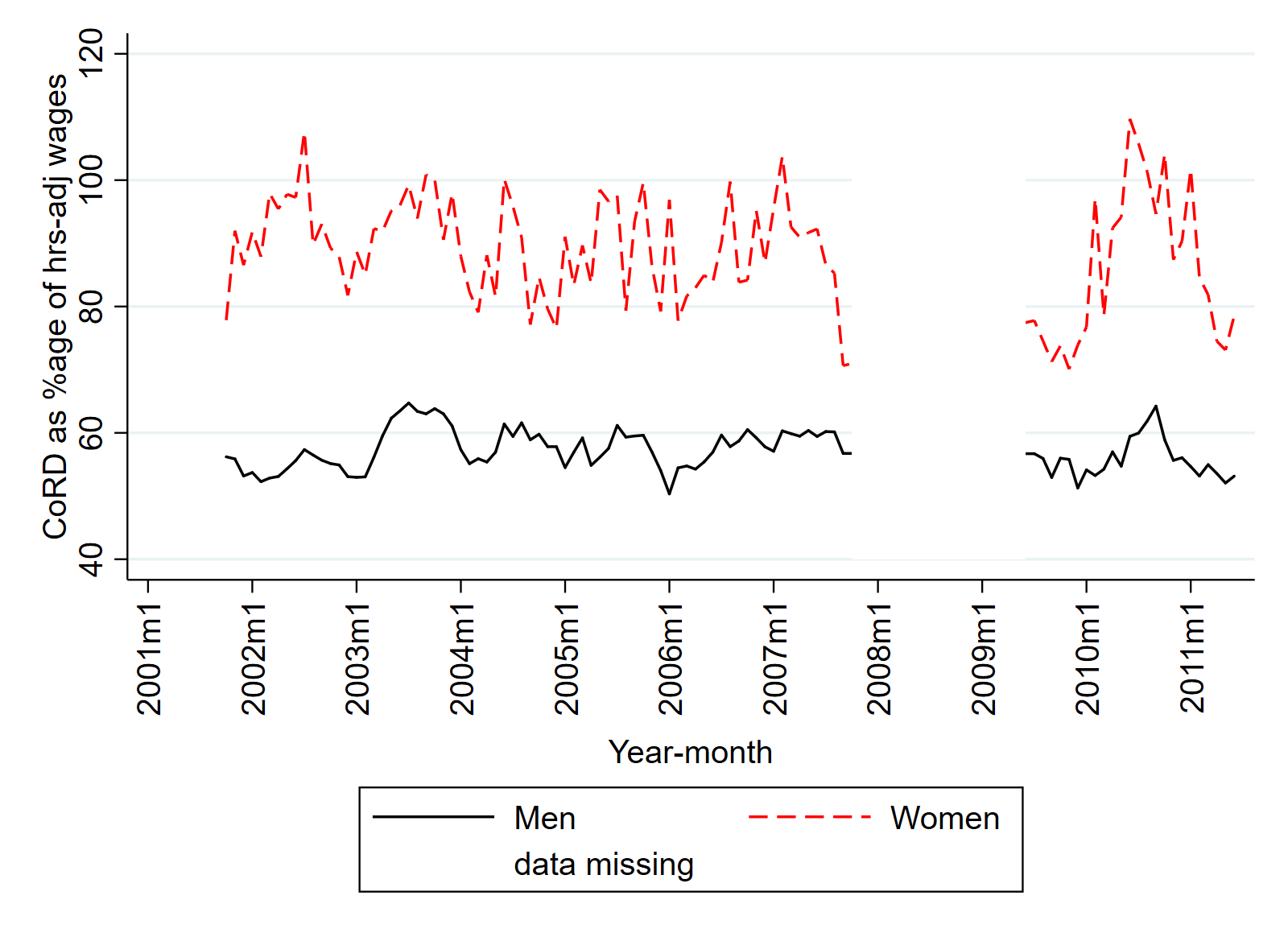


Figure A.2: Trends over time in CoRD relative to hours-adjusted unskilled labor wages, men and women


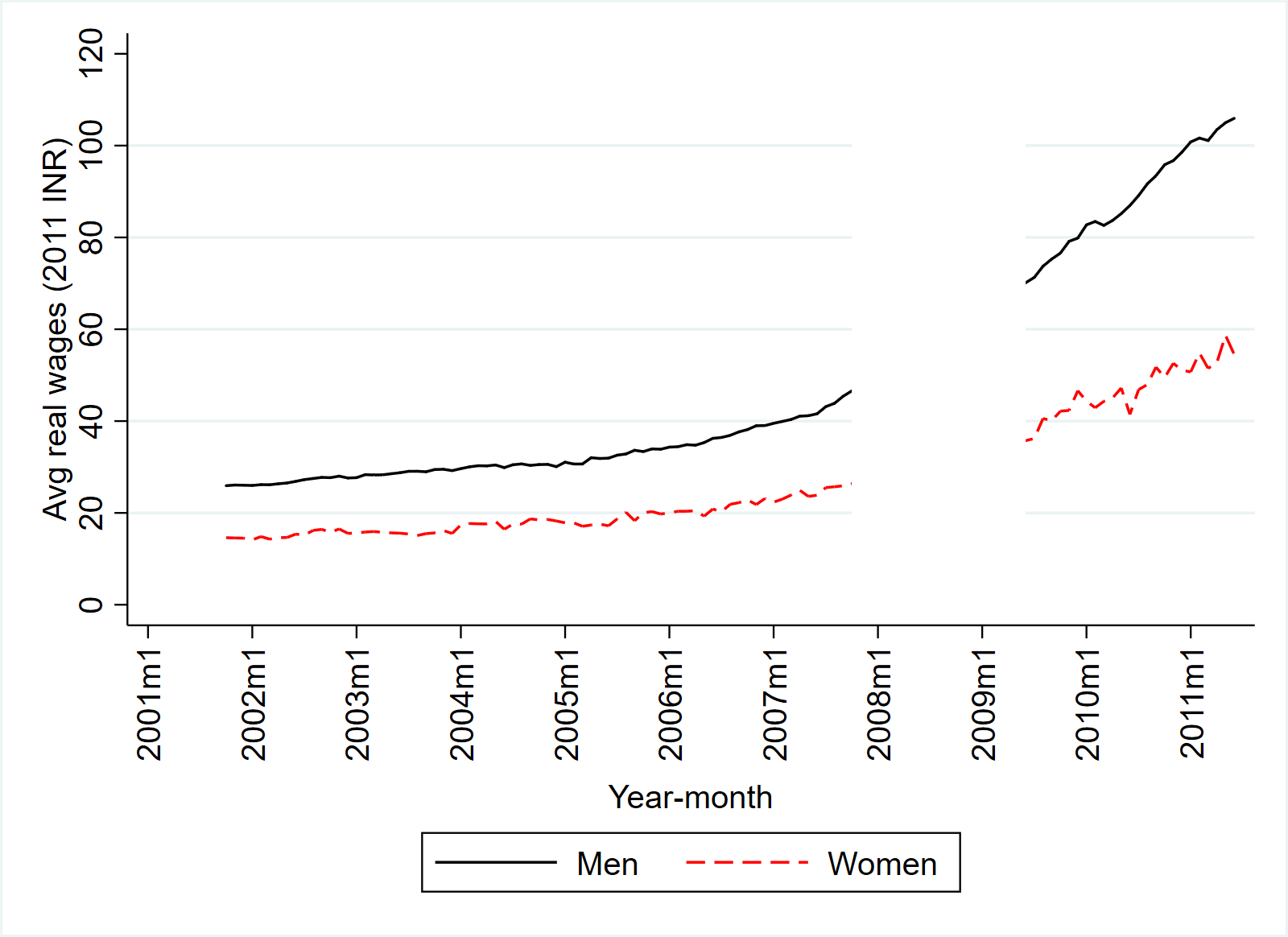


Figure A.3: Trends in hours-adjusted real wages (wage earnings) for men and women engaged in unskilled labour (2011 INR)

Figure A.4: Seasonality in log of food prices by food group, over months of the year


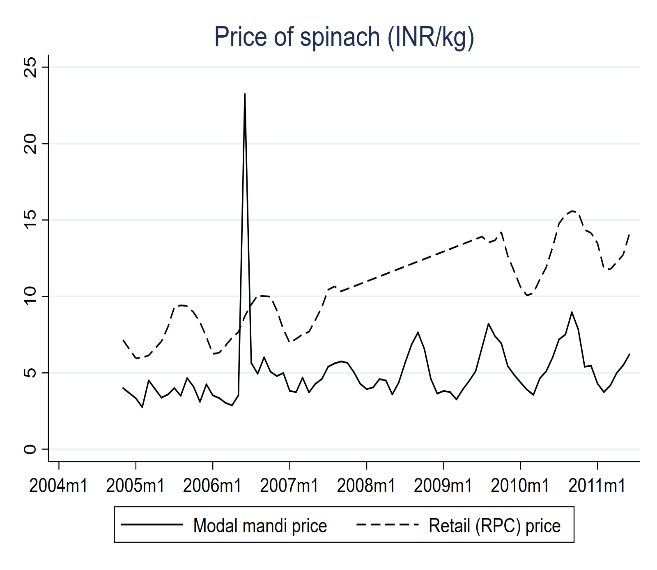

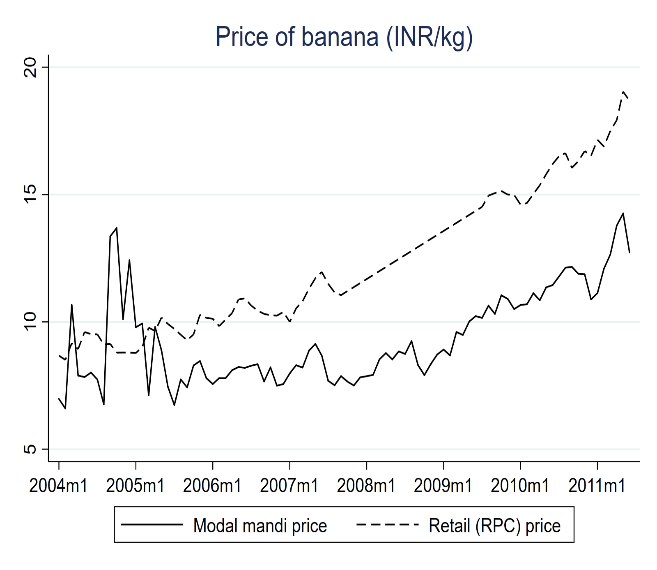

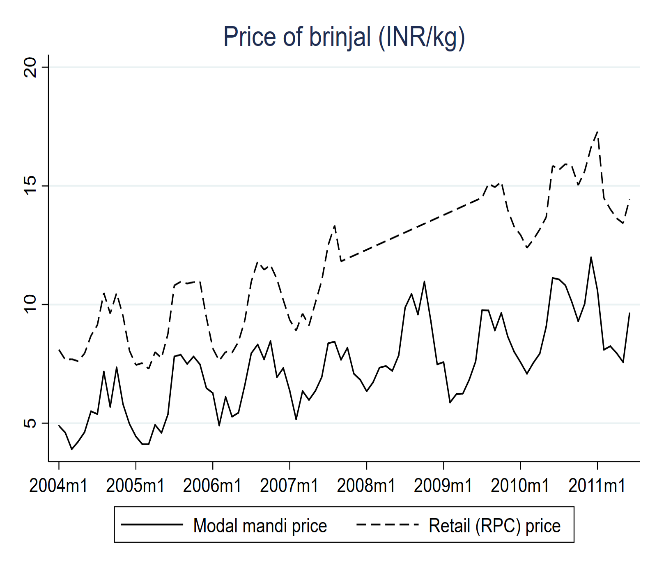

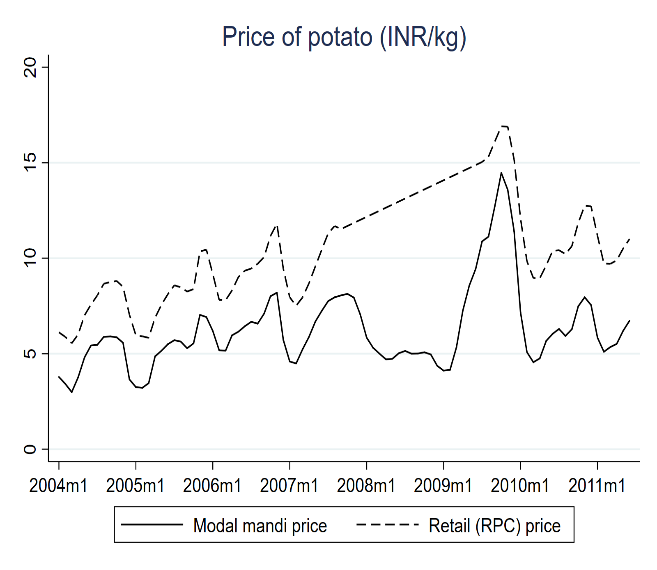

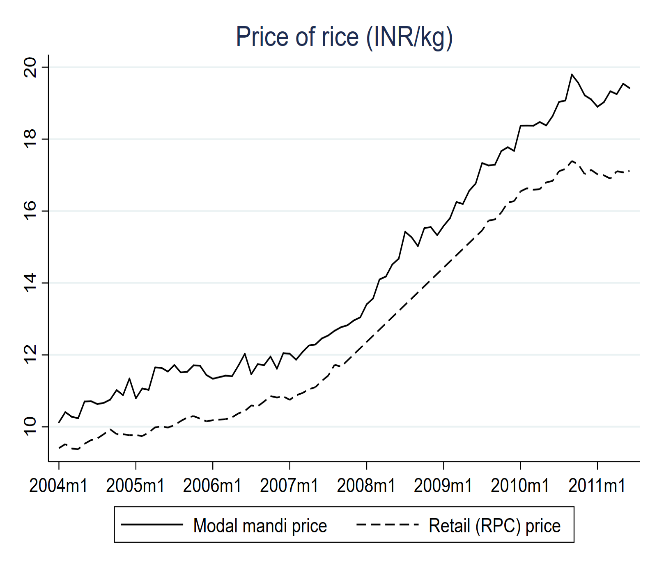

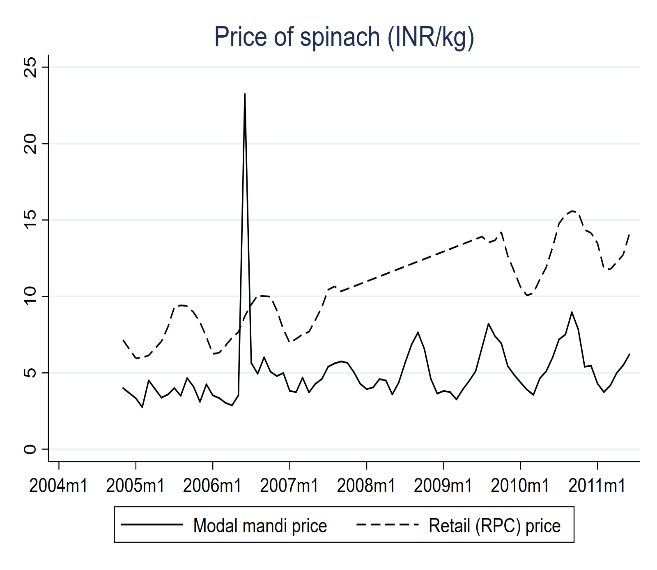

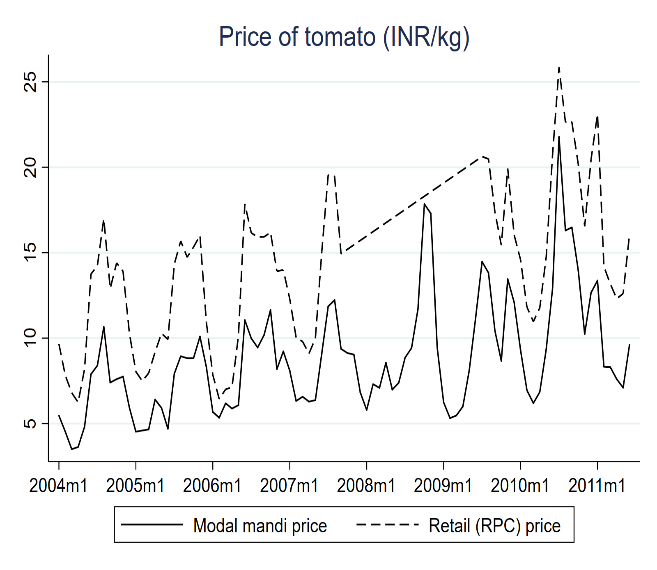

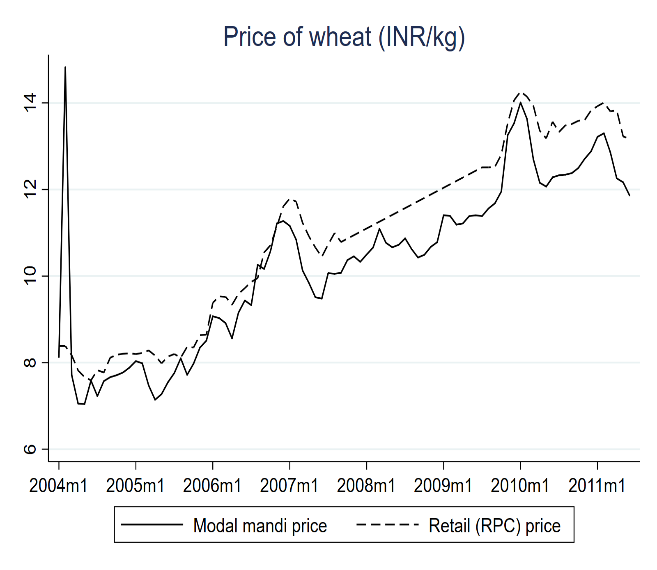

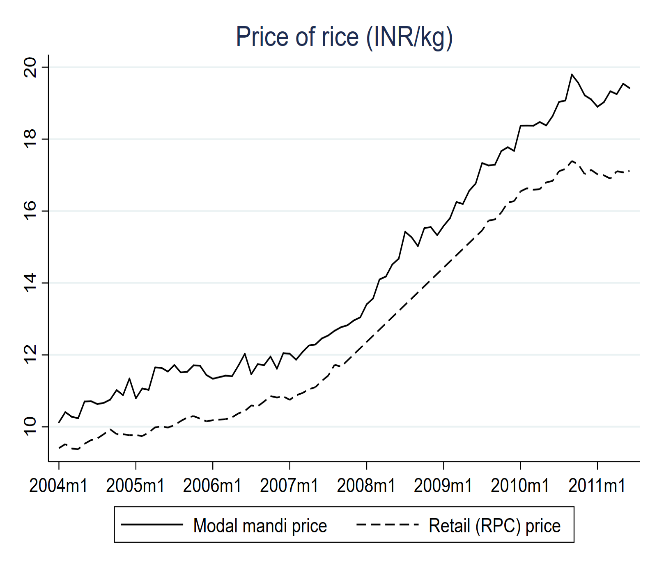

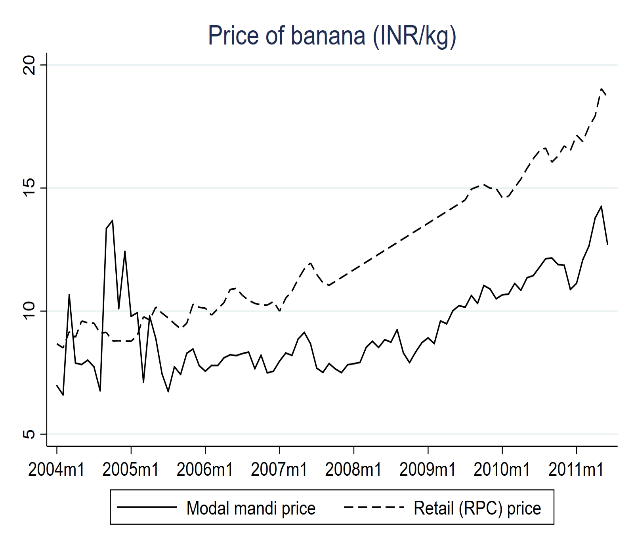

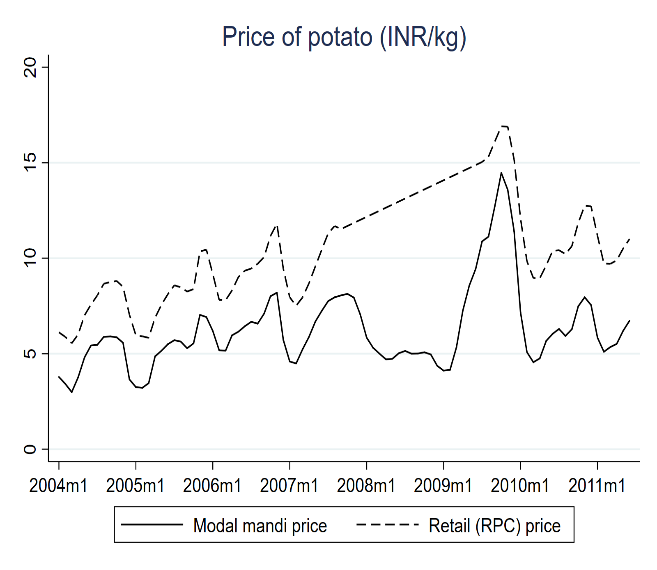

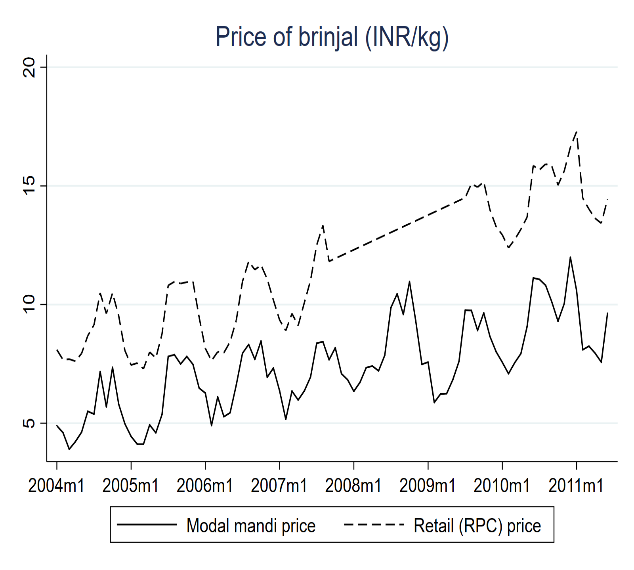


Figure A.5: Comparison of retail and wholesale prices (INR/kg) for select commodities, 2001-2011

Note: Retail (RPC) price is missing for the period 2007m10 to 2009m6; for that period the series has been completed through simple linear interpolation.
